# Acoustic Metasurface for Space‐time Reflection Manipulation

**DOI:** 10.1002/advs.202506308

**Published:** 2025-06-26

**Authors:** Yunhan Yang, Han Jia, Jiuyang Lu, Yuzhen Yang, Tuo Liu, Jun Yang, Zhengyou Liu

**Affiliations:** ^1^ Key Laboratory of Noise and Vibration Research Institute of Acoustics Chinese Academy of Sciences Beijing 100190 China; ^2^ University of Chinese Academy of Sciences Beijing 100049 China; ^3^ State Key Laboratory of Acoustics Institute of Acoustics Chinese Academy of Sciences Beijing 100190 China; ^4^ Key Laboratory of Artificial Micro‐ and Nanostructures of Ministry of Education and School of Physics and Technology Wuhan University Wuhan 430072 China; ^5^ Institute for Advanced Studies Wuhan University Wuhan 430072 China

**Keywords:** acoustic metasurface, direction‐of‐arrival estimation, space‐time modulation, waterborne sound manipulation

## Abstract

Recently, space‐time modulation has revolutionized the wave engineering technologies, providing unprecedented opportunities beyond traditional static systems. This advancement is crucial across diverse fields, ranging from non‐reciprocal transmission to wireless communication. However, the current approaches to sound modulation require bulky artificial structures and are limited in achieving space‐time‐variable sound‐matter interactions. Here, a prototype of space‐time acoustic metasurface (STAM) is proposed and implemented, consisting of a reflective piezoelectric array controlled by a field‐programmable gate array. Leveraging the spatiotemporally programmable phases of the STAM, this is experimentally achieved Doppler‐like chirp modulation and space‐time modulation with deterministic frequency and momentum shifts of waterborne acoustic waves. Furthermore, based on this flexible and efficient modulation strategy, a stochastic space‐time modulation method is introduced, showcasing its applications in single‐channel direction‐of‐arrival estimation. The proposed STAM extends the frontier of wave control and thereby lays the foundation for versatile space‐time applications involving sound.

## Graphical abstract

This article introduces a novel space‐time acoustic metasurface (STAM) to overcome the limitations of bulky structures in sound modulation. Using a programmable piezoelectric array, the STAM precisely controls waterborne acoustic waves through spatiotemporal phase modulation. Doppler‐like chirp modulation and stochastic modulation are demonstrated, with applications shown in single‐channel direction‐of‐arrival estimation, opening new avenues for wave control.

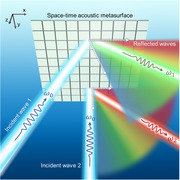

## 1 Introduction

Over the past decades, metamaterials have offered diverse medium properties unattainable in natural materials and transformed the way we engineer the wavefield. As 2D metamaterials, metasurfaces have emerged as practical tools for wave manipulation due to their facile fabrication and small sizes. Initially conceived as arrays of spatially arranged subwavelength building units (meta‐atoms),^[^ ^1^ ^]^ metasurfaces can deliberately introduce abrupt transverse phase shifts and thus enable the flexible design of light beams. This concept, known as the generalized Snell's law, has rapidly been extended to the fields of electromagnetics, acoustics, and mechanics through spatially varying the amplitudes, phases, and polarizations of meta‐atoms. Numerous demonstrations of wave manipulation are achieved with metasurfaces, such as beam shaping,^[^ ^2^, ^3^, ^4^, ^5^ ^]^ transmission enhancing,^[^ ^6^, ^7^ ^]^ nonlinear interferometry,^[^ ^8^ ^]^ and polarization imaging.^[^ ^9^, ^10^ ^]^ However, these space‐variant devices are inherently limited by Lorentz reciprocity and time‐reversal symmetry.

To overcome these constraints and explore new physics, a temporal gradient has been introduced into electromagnetic metasurfaces.^[^ ^11^ ^]^ Space‐time modulation in propagation medium has been used for nonreciprocal mode transition,^[^ ^12^ ^]^ selective wave filtering^[^ ^13^ ^]^ and rectification.^[^ ^14^ ^]^ The advent of space‐time metasurfaces with different active tunning elements incredibly extends the abilities of artificial devices to perform diverse mode conversions in free space^[^ ^15^, ^16^, ^17^, ^18^ ^]^ and leads to plenty of important phenomena, including frequency conversion,^[^ ^19^ ^]^ power combing of waves,^[^ ^20^ ^]^ and dynamic computing.^[^ ^21^ ^]^ To improve the practicability of space‐time metasurfaces, digitally coding electromagnetic metasurfaces have also been proposed with programmable space‐time states.^[^ ^22^ ^]^ This higher‐level controllability of space‐time‐coding digital metasurfaces has enabled a variety of applications in electromagnetics, including secure wireless communication,^[^ ^23^, ^24^ ^]^ doppler cloak,^[^ ^25^ ^]^ and multi‐channel transmission.^[^ ^26^, ^27^, ^28^ ^]^

Despite the rapid developments in space‐time metasurfaces for optics and electromagnetics, space‐time metasurfaces for acoustics remain in their infancy. Several time‐modulating elements, such as servo motors,^[^ ^29^, ^30^ ^]^ loudspeaker arrays,^[^ ^31^, ^32^ ^]^ tensioned membranes,^[^ ^33^ ^]^ and carbon nanotube films,^[^ ^34^ ^]^ have been proposed for acoustic metasurfaces. However, these elements generally suffer from long response time, low precision, and bulkiness, making it a challenge to achieve precise control over the frequency and momentum of acoustic waves. The lack of high‐speed and efficient modulation mechanisms further hinders advancements in sound manipulation and modern communication systems, particularly in vast underwater environments, where acoustic waves act as the sole viable option for long‐range oceanic exploration and communication.^[^ ^35^, ^36^, ^37^ ^]^

**Figure 1:**
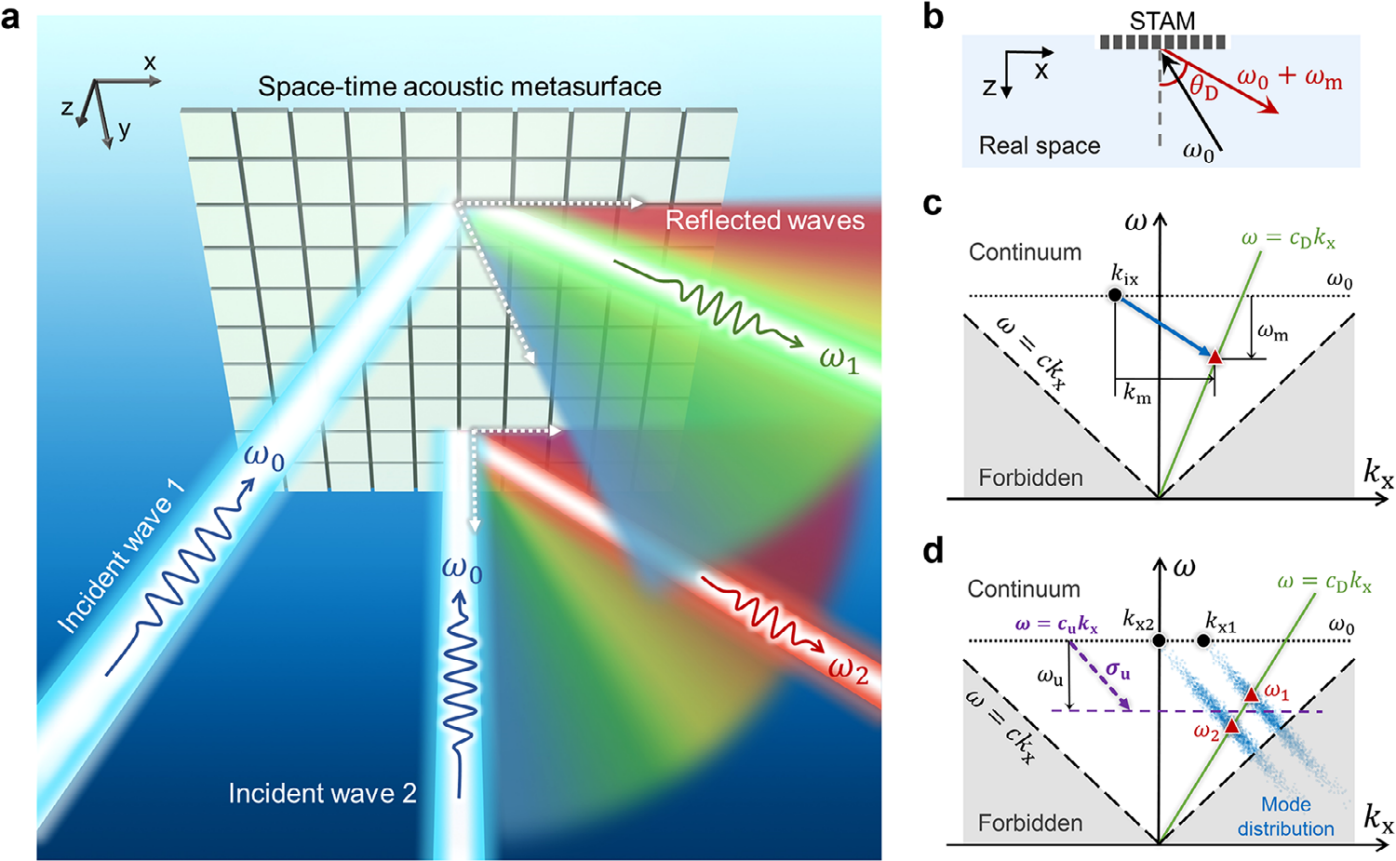
Schematic of the space‐time modulation based on the STAM. a) Real‐space representation of the underwater stochastic space‐time modulation. b) Real‐space representation of the deterministic space‐time modulation. c) Deterministic space‐time modulation depicted in the *k*_x_ −ω space. The green line indicates the spectrum detected by a stationary detector in the direction of θ_D_. The blue arrow denotes the space‐time modulation with deterministic frequency (ω_m_) and momentum (*k*_m_) shifts. d) Stochastic space‐time modulation depicted in the *k*_x_ −ω space. The purple arrow represents the unit vector of space‐time modulation σ_u_, while the blue points illustrate the reflected modes. The mode distributions constructed by blue points display certain bandwidths that reflect realistic outcomes due to modulation imperfections.

Here, we propose a space‐time acoustic metasurface (STAM) to realize full control of reflection phases in the spatial and temporal domains. As shown in **Figure** **1**a, the STAM comprises an array of piezoelectric meta‐atoms, each independently controlled by a field‐programmable gate array (FPGA). Through modulating space‐time phase gradients, the Lorentz reciprocity and frequency conservation of the reflection process are broken, thereby enabling versatile mode conversions. As an experimental demonstration, we simultaneously manipulate both the frequency and momentum of the reflection waves to achieve Doppler‐like chirp modulations and deterministic space‐time modulations. We further develop a method based on stochastic space‐time modulation to guide reflected modes into elaborately designed distributions in the frequency‐momentum space. Utilizing the bridging effect of these distributions, we realize the real‐time direction‐of‐arrival (DOA) estimation simply from the observed spectra. The proposed STAM represents a general and flexible approach to sound manipulation and paves the way for broader applications in space‐time wave modulation.

## 2 Results and Discussion

### 2.1 Theory of Space‐Time Modulation

As illustrated in Figure 1a, the STAM is positioned facing underwater to reflect acoustic waves. The STAM exhibits engineered reflection phase distributions in both temporal and spatial domain, introducing frequency shifts and transverse momentum shifts along the x‐direction. In the time domain, we assume that the modulation period is significantly longer than that of the incident waves, indicating that the modulation frequency is much lower than the frequency of the incident waves, thereby justifying the use of slow temporal modulations.^[^ ^12^ ^]^ In real space, the reflection process can be described as

(1) $$p_{r}\;\left(x,z,t\right)=p_{i}\;\left(x,z,t\right)\cdot \Gamma \cdot e^{j\varphi \left(x,t\right)}$$

where *p*_i_, *p*_r_, Γ, and φ denote the incident wave, reflected wave, reflection amplitude, and reflection phase function, respectively. In our approach, the reflection process is implemented in the form of phase modulation with a uniform reflection amplitude (Γ  =  1). The STAM imparts external momentum and frequency shifts to incident waves by dynamically changing the reflection phases of each meta‐atom. Given that reflection is a time‐domain product operation, its Fourier transform in the mode space is related to a convolution operation. In other words, the phase function acts as a convolution kernel, enabling mode shifts in the frequency‐momentum space.^[^ ^3^, ^38^, ^39^ ^]^ For a deterministic space‐time modulation with momentum and frequency components (*k*_m_ and ω_m_), the phase function is expressed as φ (*x*, *t*) = ω_m_ *t* − *k*_m_ *x*. When an acoustic plane wave $p_{i}=P_{0}\;e^{j(\omega_{0}t-k_{\mathrm{ix}}x+k_{\mathrm{iz}}z)}$ impinges on the STAM with a certain transverse wavevector and frequency (*k*_ix_,ω_0_), as indicated by the black dot in Figure 1c, the reflected wave is modulated to $p_{r}=P_{0}\;e^{j[\left(\omega_{0}+\omega_{m}\right)t-\left(k_{\mathrm{ix}}+k_{m}\right)x-k_{\mathrm{rz}}z]}$ (harmonic analysis provided in Note S1, Supporting Information). Here, the transverse and normal momentum components of incident wave are denoted as *k*_ix_ and *k*_iz_, respectively. *k*_ix_ < 0 presents an incident wave propagating in the negative x‐direction. This modulation corresponds to a mode shift to (*k*_ix_ + *k*_m_,ω_0_ + ω_m_), represented by the red triangle in Figure 1c, where *k*_ix_ + *k*_m_ > 0 is used for an instance. The real‐space representation is shown in Figure 1b, illustrating an anomalous reflection characterized by a reversal in the transverse propagation direction and a change in frequency. The z‐component wavevectors of incident and reflected waves are given by $k_{\mathrm{iz}}=\sqrt{\omega_{0\;}^{2}/c^{2}-k_{\mathrm{ix}}^{2}}$ and $k_{\mathrm{rz}}=\sqrt{{\left(\omega_{0}+\omega_{m}\right)}^{2}/c^{2}-{\left(k_{\mathrm{ix}}+k_{m}\right)}^{2}}$, respectively, according to the dispersion relation; *c* denotes the speed of sound in water.

As a result of convolution, the reflected modes preserve the information of the incident waves and can be modified using a pre‐designed phase function of the STAM. A finely designed phase function enables the application of the STAM in the single‐channel DOA estimation, a critical technique in modern communication systems for target detection.^[^ ^24^, ^40^, ^41^, ^42^ ^]^ To exploit this, we propose the stochastic space‐time modulation creating quasi‐continuous reflection distributions in the *k*_x_ −ω space. In this context, we define a stochastic phase function φ (*x*, *t*) =  ξ(*t*) (ω_u_ *t* − *k*_u_ *x*) =  ξ(*t*)ω_*u*_(*t* −1/*c_u_x*), where $\xi \;\left(t\right)=\sum_{l}\xi_{l}U_{l}\;\left(t\right)$. Here, $\xi_{l}\in R$ represents the coefficients following a designed stochastic distribution Ξ, and *U_l_* (*t*) is a pulse function defined as $U_{l}\;\left(t\right)=\{\begin{array}{c} 1,\;(l-1)T<t<lT\\ 0,\;otherwise\; \end{array}$; *T* denotes the pulse width of *U_l_*, corresponding to the duration of a deterministic space‐time modulation with single mode conversion. From the perspective of *k*_x_ −ω space, the stochastic function ξ(*t*) operates on the unit vector of space‐time modulation σ_u_ = ω_u_ (1/*c*_u_,1), which is utilized to determine the slope and length of mode conversion (depicted as the purple dashed arrow in Figure 1d). Consequently, the mode conversion length is variable, while the relation between time and spatial gradient is not influenced. This interaction leads to mode conversions with a constant slope but a temporally varying length, forming line‐shape patterns over a larger timescale. Specifically, *c*_u_ = ω_u_ /*k*_u_ represents the characteristic speed of the modulation related to the slope. ω_u_ signifies the unit frequency shift and serves as the reference length for mode conversion. It is determined by the central angle of the desired range (typically normal incidence with *k*_ix_ =  0 and ω  = ω_0_ ), as well as the intersections along the characteristic speed of the modulation. Given a plane wave incidence (*k*_ix_,ω_0_), the reflected wave modes distributed in the *k*_x_ −ω space are given by:

(2) $$\left(k_{\mathrm{ix}},\omega_{0}\right)*\;\left(\Xi \cdot \sigma_{u}\right)=\left(k_{\mathrm{ix}}+\Xi \cdot \frac{\omega_{u}}{c_{u}},\;\omega_{0}+\Xi \cdot \omega_{u}\right)$$

where Ξ represents the distribution of ξ_*l*_, and * denotes the convolution operation. We consider the case that the incident waves have the same frequency but two different transverse wavevectors (*k*_x1_,ω_0_) and (*k*_x2_,ω_0_), which are depicted as black dots in Figure 1d. Under Gaussian‐distribution stochastic modulation ξ, the incident modes are transformed into two parallel distributions of reflected modes (blue dots in Figure 1d), manifesting as two blue bands with the same slope. These reflected mode distributions center at the frequency of ω_0_ + ω_u_ and covers a broad range of angles. Regardless of the specific locations of *k*_x1_ and *k*_x2_, they can be estimated, as long as their broad mode distributions intersect with the detection line. The green line in Figure 1d represents the detected spectrum line at an angle of θ_D_ relative to the STAM, and its slope can be given as *c_D_* =  *c*/sin θ_*D*_. As these distributions intersect with the detected spectrum line, two separate intersections emerge (red triangles), showing as two distinct frequencies in the detected spectrum (ω_1_ and ω_2_). Owing to the broad stochastic distribution, a wide range of incidences with diverse wavevectors can be detected at a given angle. This is different from deterministic space‐time modulation scenarios, which require priori knowledge of both momentum and frequency shifts for observation (Figure 1c). To pursue a clear image, we illustrate the real‐space representations of the stochastic modulation in Figure 1a. The two rainbow‐like regions represent all the reflected waves from two different incident angles. These distributions vary with incident angle at the same detection angle: a higher frequency (ω_1_) occurs for oblique incidence, while a lower frequency (ω_2_) is observed for normal incidence. In this way, we develop an intuitive DOA estimation strategy based on the one‐to‐one correspondence between the incidence angle θ_i_ and the measured frequency ω_c_ (see detailed calculation in Note S2, Supporting Information):

(3) $$\theta_{i}\;\left(\omega_{c}\right)=\sin^{-1}\left[\frac{c}{c_{u}}-\frac{\omega_{c}}{\omega_{0}}\left(\frac{c}{c_{u}}+\sin\theta_{D}\right)\right]$$

### 2.2 Experimental Demonstration

**Figure 2:**
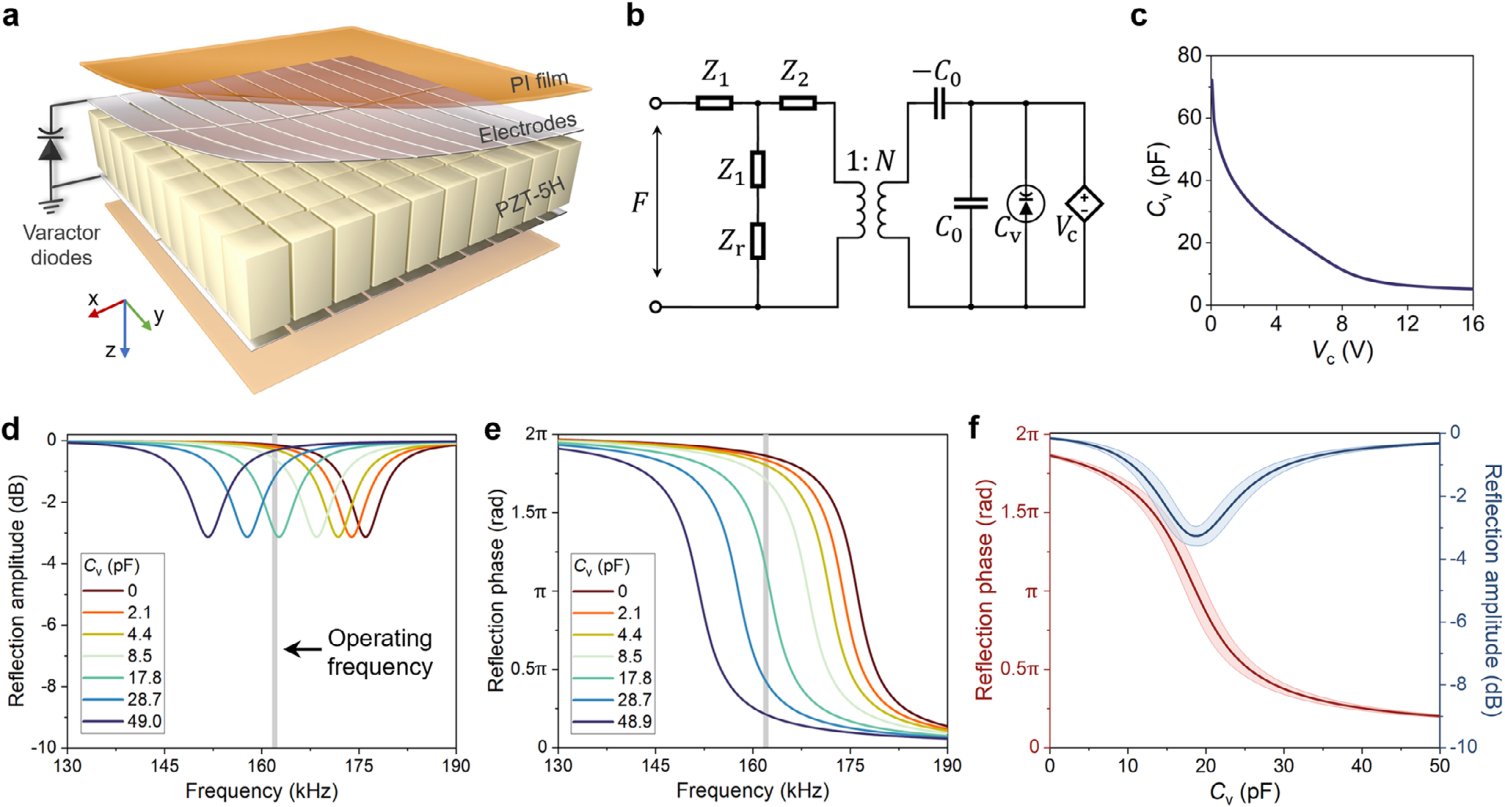
Design and characterization of the STAM. a) Schematic of the STAM comprising an array of meta‐atoms. Details are given in the Experimental Section. b) Equivalent circuit of a meta‐atom. c) Measured average curve of the varactor diode capacitance *C*_v_ for individual meta‐atom relative to the bias voltage *V*_c_. d) Representative reflection amplitudes of the meta‐atoms in the 16th channel under different external capacitances. The grey line marks the operating frequency of 162 kHz. e) Representative reflection phases of the meta‐atoms in the 16th channel under different external capacitances. f) Reflection phases (red) and amplitudes (blue) of all meta‐atoms as functions of external capacitances at the operating frequency. The solid lines represent average values and shaded regions indicate error bands.

To implement space‐time modulations for waterborne sound, we take an array of 10 × 10 piezoelectric lead zirconate titanate 5H (PZT‐5H) rectangular pillars to construct the STAM (**Figure** **2**a). All the piezoelectric pillars are polarized along the thickness direction (z‐direction) and spatially separated to support independent resonances. In the x‐direction, all 10 columns are electrically isolated, whereas in the y‐direction, each group of 5 pillars shares common electrodes. These 5 piezoelectric pillars sharing same electrodes constitute one channel of the STAM (i.e., 20 channels in total). Two channels with the same x‐coordinate yield identical reflection phases, thereby confining the phase modulation to the x‐direction. Polyimide (PI) films isolate the electrodes from the external environment (fabrication details are provided in Experimental Section). To achieve programmable reflection phases, each piezoelectric pillar is equipped with varactor diodes, forming a meta‐atom. The equivalent circuit of the meta‐atom is depicted in Figure 2b. The capacitances *C*_v_ of the varactor diodes in each channel are independently modulated by a time‐varying bias voltage *V*_c_ from a FPGA. Figure 2c shows the measured capacitance *C*_v_ curves for an individual meta‐atom. Generally, an increase in the bias voltage *V*_c_ results in a decrease in *C*_v_, spanning from 72 to 5 pF.

Figures 2d,e present the semi‐analytic reflection spectra of a typical meta‐atom derived from the equivalent circuit model using a scattering matrix method (see detailed expressions and experimental process in Experimental Section and Note S3, Supporting Information). We consider the STAM interfacing with water on one side and air on the opposite side. Under this condition, varying the external capacitances *C*_v_ induces a shift in the resonance frequency of the meta‐atom, indicated by the dissipation‐induced reflection dips in Figure 2d and π‐shifts in Figure 2e, respectively. As *C*_v_ varies from 0 to 50 pF, the resonance frequency shifts from 178 to 151 kHz. Figure 2f presents the semi‐analytic reflection amplitudes |Γ| and phases φ for all meta‐atoms at the operating frequency of *f*_0_ =  162 kHz. A nearly full 2π phase shift with small amplitude varying is observed as *C*_v_ changes, confirming the high‐resolution phase modulation capabilities of the meta‐atom. By incorporating the control voltage *V*_c_ and capacitance *C*_v_, we derive the desired reflection phases φ(*x*, *t*) as a function of the time‐varying *V*_c_ for each meta‐atom. A universal long‐sequence discretization strategy is then employed to encode the entire period of the phase functions into discrete‐time states (see Note S1 for details, Supporting Information), enabling the implementation of tens of thousands of distinct programmable reflection phases. This strategy overcomes the limitations of short coding sequences with few elements used in deterministic space‐time modulation,^[^ ^22^, ^43^ ^]^ unlocking the potential for non‐uniform and stochastic space‐time modulations.

**Figure 3:**
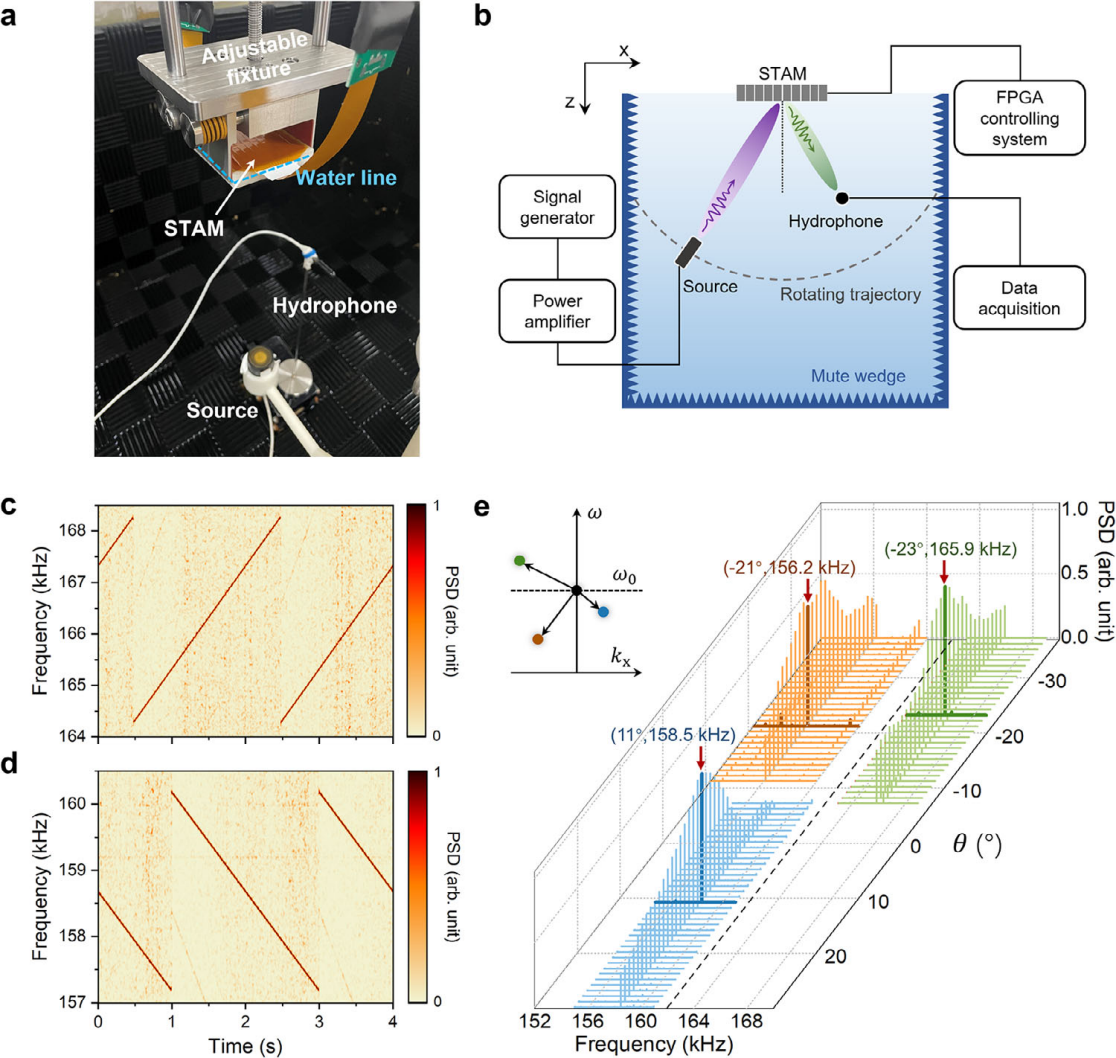
Experiment setup and results of space‐time modulations. a,b) Photograph (a) and schematic (b) of the experimental setup. c) Normalized PSD spectrogram of reflection in the first chirp modulation experiment with normal incidence and reception. The chirp modulation parameters are set to ω_s_ =  2.3 × 2π kHz, and *u*  =  4 × 2π kHz, periodically modulating the target reflection frequency from 164.3 kHz to 168.3 kHz within 2 s. d) Normalized PSD spectrogram of reflection in the second chirp modulation experiment with normal incidence and reception. The chirp modulation parameters are set to ω_s_ =   −1.8 × 2π kHz, and *u*  =   −3 × 2π kHz, periodically modulating the target reflection frequency from 160.2 kHz to 157.2 kHz within 2 s. e) Measured normalized PSD for three different space‐time modulations. The mode conversion slopes and frequency shifts ($c_{\mathrm{st}}^{\left(n\right)}$ and $\omega_{\mathrm{st}}^{\left(n\right)}$) are set as (−*c*/9, −3.5 × 2π kHz),(−*c*/16, +3.9 × 2π kHz), (*c*/10, −5.8 × 2π kHz), corresponding to the blue, green, and brown curves, respectively. The bold lines denote spectra in the target reflection directions and the red arrows indicate the coordinates of measured peaks. The dashed line denotes the operating frequency. *f*_0_ =  162 kHz. The inset depicts the related mode conversion processes in the *k*_x_ −ω space.

The experimental setup for demonstrating the space‐time modulation capabilities of the STAM is illustrated in **Figures** **3**a,b as a photograph and a schematic, respectively. Measurements are conducted in an anechoic tank, with the STAM hung from an adjustable fixture and partially immersed in water (see the overall setup in Figure S5, Supporting Information). We use a piezoelectric transducer as the sound source and a hydrophone as the detector to capture the reflection spectrum (see details in the Experimental Section). We first conduct Doppler‐like chirp modulation experiments by designing different chirp functions to achieve long‐duration reflection frequency control. In experiments, both the source and the hydrophone are positioned vertically to the center of the metasurface. The source continuously emits a sinusoidal wave at the operating frequency *f*_0_ and a linearly‐varying frequency modulation is applied to the STAM. The chirp modulation phase function, emulating the Doppler effect under relative movement, is given by $\varphi \;\left(t\right)=\omega_{s}\;t+\frac{1}{2}ut^{2}$, where ω_s_ is the static angular frequency shift and *u* is the Doppler shift coefficient. The scattering process is described by $\left(k_{\mathrm{ix}},\omega_{0}\right)\to \left(k_{\mathrm{ix}},\omega_{0}+\omega_{s}+\frac{1}{2}ut\right)$, where *k*_ix_ =  0 and ω_0_ =  2π*f*_0_. The phase function is configured to repeat every 2 s with 40000 discrete reflection phases, each lasting 50 us. Figures 3c,d display the normalized power spectral density (PSD) spectrograms in the time‐frequency domain from the two chirp modulation experiments. In Figure 3c, an explicit periodic frequency shift is observed from 164.3 to 168.3 kHz and then back to 164.3 kHz. Figure 3d shows another result where the reflected wave is repeatedly shifted from 160.2 to 157.2 kHz. Both experiments demonstrate precise reflection frequency shifts and high signal‐to‐noise ratios, confirming the capabilities of the STAM for accurate frequency modulation across a large number of programmable reflection states.

Next, we simultaneously control the spatial and temporal variations of the phase function to achieve three different space‐time modulations, each characterized by unique mode conversion slopes and frequency shifts in the *k*_x_ −ω space ($c_{\mathrm{st}}^{\left(n\right)}$ and $\omega_{\mathrm{st}}^{\left(n\right)}$, where *n*  =  1, 2, 3). In this setup, a stationary source generates a normal incident wave, while a hydrophone positioned on the collaborative robot captures the reflected wave distributions with a rotation angle of 1°. Through the design of *c*_st_ and ω_st_, we aim to direct the reflected waves to (11.2°, 158.5 kHz), (−22.6°,  165.9 kHz),  and (−21.0°,  156.2 kHz). The mode conversions of these reflection processes are indicated by the blue, green, and brown colors in the inset of Figure 3e, respectively. Figure 3e illustrates the measured spectra in the three experiments. The bold lines depict the reflection spectra in the target directions while the red arrows indicate the measured peak frequencies, with corresponding coordinates marked above. Distinct deflection angles indicate the transverse momentum shifts generated by the STAM, as the incident waves do not possess any transverse momentum components. The remarkable agreement between the desired reflected modes and the measured peaks confirms the STAM's capability for flexible and precise control over both the frequency and momentum of reflected waves. The spatial‐domain lineshapes, with certain beamwidths, are related to the far‐field directivities of the STAM.

### 2.3 Stochastic Space‐Time Modulation For Single‐Channel DOA Estimation

**Figure 4:**
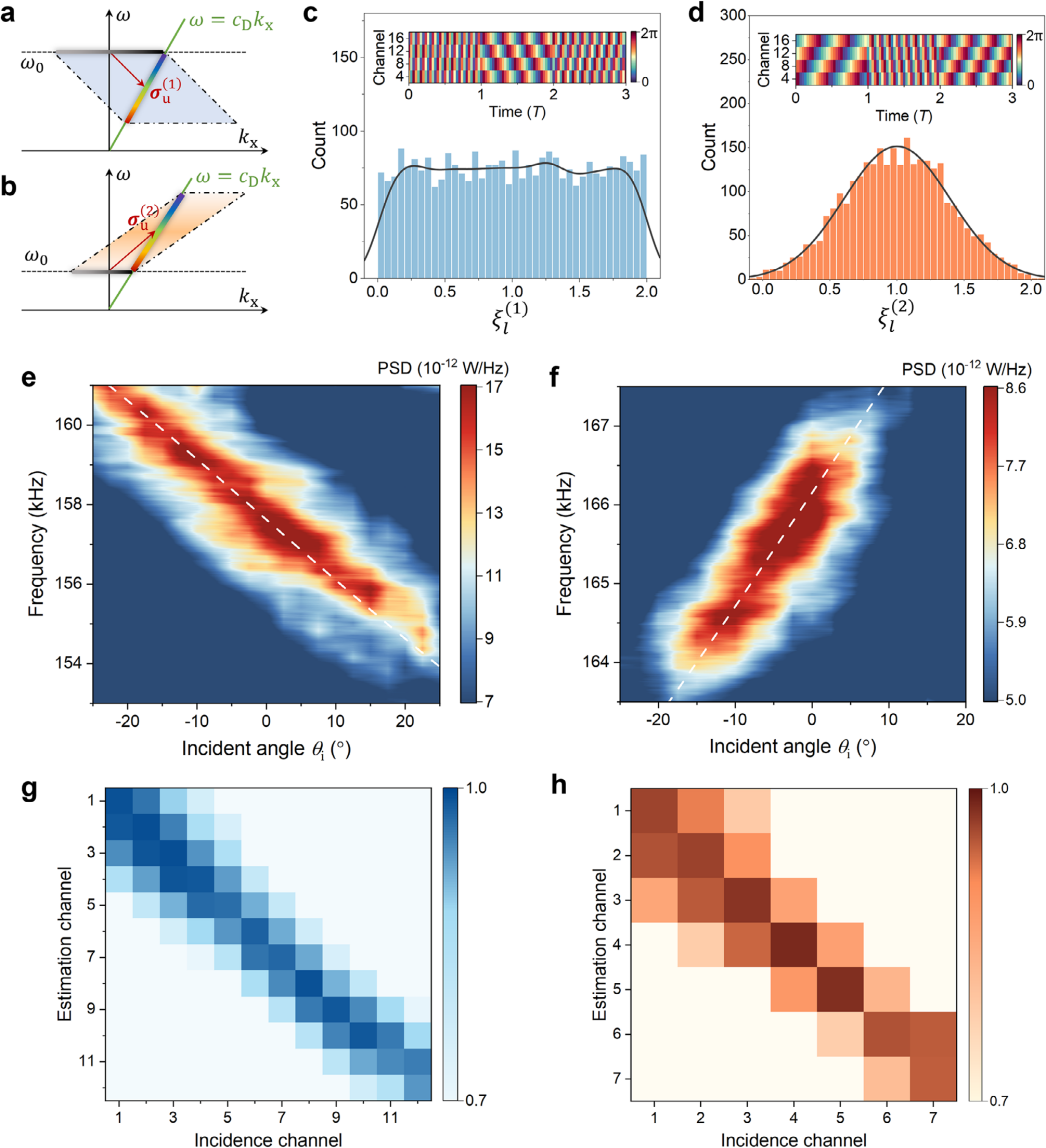
Experimental demonstration of the stochastic space‐time modulation. a, b) Schematics of down‐conversion (a) and up‐conversion (b) stochastic space‐time modulation processes in the *k*_x_ −ω space. The bold grey lines represent incident modes with a series of angles, whereas the rainbow lines signify the corresponding detected frequencies at the angle of 30°. The blue (a) and orange (b) regions illustrate all possible distributions of reflected modes under uniform (down‐conversion) and Gaussian (up‐conversion) stochastic modulations, with color intensity reflecting the mode density. Red arrows denote the unit mode conversions, with parameters set as $\omega_{u}^{\left(1\right)}=-4.4\times 2\pi$ kHz, $c_{u}^{\left(1\right)}=-\frac{1}{18}c$ (a) and $\omega_{u}^{\left(2\right)}=4.2\times 2\pi$ kHz, $c_{u}^{\left(2\right)}=\frac{1}{20}\;c$ (b). c, d) Distributions of the stochastic modulation functions: $\xi_{l}^{\left(1\right)}$ follows a uniform distribution (c) and $\xi_{l}^{\left(2\right)}$ follows a Gaussian distribution (d). The dark lines illustrate the distribution envelopes. Insets depict the reflection phases of the first three single space‐time modulations at channels 4, 8, 12, and 16, each lasting 1.5 ms (*T*  =  1.5 ms) e, f) Measured reflection spectra of down‐conversion (e) and up‐conversion (f) modulations at different incident angles. White dashed lines denote the theoretical prediction frequencies from intersection coordinates. g, h) Measured correlation coefficient matrices of down‐conversion (g) and up‐conversion (h) modulations, with ranges of [− 20°, 7.5°] and [− 15°,  5°], respectively. Each channel represents a 2.5° angle.

Building on the validated space‐time modulation capabilities of the STAM, we perform two stochastic space‐time modulation experiments for DOA estimation, utilizing distinct phase functions $\varphi^{\left(n\right)}\;\left(x,t\right)=\xi^{\left(n\right)}\;\left(t\right)\omega_{u}^{\left(n\right)}\left(t-\frac{1}{c_{u}^{\left(n\right)}}x\right),\;n=1,2$. The modulation unit vectors are set as $\sigma_{u}^{\left(n\right)}=\omega_{u}^{\left(n\right)}\;\left(\frac{1}{c_{u}^{\left(n\right)}},1\right),\;n=1,2$, with opposite slope signs (indicated by red arrows in Figures 4a,b). The lengths of the unit vectors are determined by the normal incident mode and their respective intersections. This configuration anticipates identical incidence but with different frequencies in observation. The first modulation demonstrates a down‐conversion momentum‐to‐frequency mapping ($c_{u}^{\left(1\right)}<0$), while the second employs an up‐conversion mapping ($c_{u}^{\left(2\right)}>0$), as presented in **Figures** **4**a,b, respectively. In both experiments, a stationary detector is positioned at a 30° angle relative to the STAM, resulting in a detected spectrum line slope of *c*_D_ =  *c*/sin 30°. The stochastic functions ξ^(*n*)^(*t*), *n*  =  1, 2 are designed to follow two kinds of distributions: $\xi_{l}^{\left(1\right)}$ are sampled from a uniform distribution *U*(0, 2), while $\xi_{l}^{\left(2\right)}$ are sampled from a Gaussian distribution *r* × *N*(1, *I*) which centered at 1 (Figures 4c,d). Here *I* is the identity matrix, and *r* represents the amplitude of the random function, which is set as 0.4 to make most samples located within [0,2]. The uniform distribution generates an averaged energy profile in the reflected area (Figure 4c), resulting in a broader estimation range. In comparison, the Gaussian distribution has a more concentrated energy profile (Figure 4d). This concentration results in significantly higher counts of reflected modes within the range of [0.5,1.5], substantially increasing the probability of detecting incident modes near the central momentum and enhancing estimation accuracy at angles close to normal incidence. These varying values of coefficients ξ_*l*_ influence the phase shift velocities in both spatial and temporal domains. As illustrated in the insets of Figure 4c,d, the stochastic nature of ξ_*l*_ results in unique phase shift patterns within each space‐time modulation period *T*. Figures 4e,f illustrate the measured spectra for varying incident wave directions with 2.5° increments under the two stochastic modulations (see Note S5 for measurement procedure, Supporting Information). Explicit frequency shifts with incident angles are observed across ≈50° for the down‐conversion modulation (Figure 4e) and 30° for up‐conversion modulation (Figure 4f). These measured frequency components indicate the reflected modes deflected to an angle of 30°, illustrating the effect of both frequency and momentum shifts. A high degree of consistency is evident between the experimental observations and theoretical frequencies (calculated using Equation 3 and depicted by white dashed lines). To assess the accuracy of the proposed strategy, we calculate the correction matrices^[^ ^9^, ^44^ ^]^ in Figures 4g,h to evaluate the correlation coefficients between the measured results and the theoretical Lorentzian lineshapes (Note S6, Supporting Information). The diagonal terms act as the maximum values of the matrix, indicating the estimated incidence channels exactly align with the actual incidence channels. These experiments confirm the effective and programmable capabilities of the STAM for DOA estimation. It is worth noting that, as demonstrated by the experimental results, σ_u_ determines the mapping relation between the incident wavevector and observed frequency, thereby serving as a key to decode the spectrum. Without the knowledge of σ_u_, even if an eavesdropper acquires the same spectra, the DOA information remains inaccessible, which ensures the security of the estimation process.

**Figure 5:**
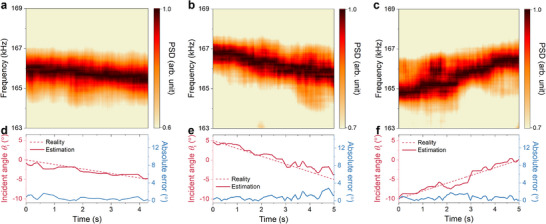
Real‐time DOA estimation of a moving source. a–c) Measured PSD spectrograms of the real‐time estimations of a moving source from 0° to ‐5° in 4.3s (a); from 5° to ‐5° in 5s (b); ‐10° to 0° in 5s (c). d‐f) The realistic (red dashed lines) and estimated (red solid lines) directions of the moving sources from 0° to ‐5° d); from 5° to ‐5° e); ‐10^○^ to 0° f). Blue solid lines are the absolute errors of the estimation.

Finally, we present real‐time DOA estimations for a moving source to test the practicability of the proposed strategy. According to the principles of stochastic processes, the random distribution Ξ remains valid even when examined on a rationally small timescale, thus allowing real‐time applications. We employ the Gaussian‐distribution modulation with $\sigma_{u}^{\left(2\right)}=\omega_{u}^{\left(2\right)}\;\left(\frac{1}{c_{u}^{\left(2\right)}},1\right)$ to conduct experiments, aiming for higher time resolution near the normal direction as previously mentioned. The moving source is mimicked by a transducer rotated at a uniform speed by the collaborative robot, while the hydrophone is fixed at a 30° angle, aligning with the *k*_x_ −ω relation in Figure 4b. Three rotations of the source are implemented: (I) 0° to ‐5° in 4.3s; (II) 5° to ‐5° in 5s; (III) ‐10° to 0° in 5s. The stochastic modulation is performed persistently as the incidence rotates. **Figures** **5a‐c** present the PSD spectrograms recorded during these experiments, showing clear frequency shifts with different starting and ending points. We extract the time‐varying peak frequencies and calculate the estimated directions using Equation 3. Figures 5d‐f illustrate both the realistic trajectories of the moving source and the corresponding estimations, showing a close alignment with absolute errors generally within 2°. In these experiments, the estimations are simply performed using the peak frequencies and a relatively high real‐time estimation accuracy is obtained at the meantime. A more concentrated distribution of Ξ and a larger array with additional meta‐atoms could further improve efficiency and accuracy of the estimation (see Note S1, Supporting Information), resulting in a narrower beam width and more precise momentum shifts.

## 3 Conclusion

To summarize, we have developed a prototype of STAM featuring diverse space‐time modulations in reflection. Leveraging the rapid response of piezoelectric meta‐atoms and the high programmability of FPGA, our STAM achieves high‐accuracy frequency and momentum shifts of reflected modes. By sweeping among reflection phases, the STAM generates linear frequency shifts that mimic the Doppler effect and implements deterministic space‐time modulations. The long‐duration phase control further enables stochastic space‐time modulation for wave detection. Unlike traditional real‐time DOA estimation systems or other metasurface‐based methods,^[^ ^24^, ^41^, ^45^ ^]^ which often face cost challenges due to the need for multiple receivers and complex real‐time processing algorithms, our proposed stochastic method leverages the inherent mode conversion characteristic of space‐time modulation. This approach establishes a direct mapping from the incident wavevector to the observed frequency, significantly reducing both complexity and hardware costs. Leveraging a long‐sequence discretization strategy, the complex space‐time varying phase functions can be readily encoded into discrete‐time state sequences, significantly reducing the design complexity and enhancing design flexibility. This method, rooted in general physical principles, can be easily extended to other wave systems, such as optics, electromagnetics, and mechanics, to enable multifunctional reflection control and detection. The proposed STAM opens new pathways for full degree‐of‐freedom sound propagation control and highlights significant potential for future ocean exploration, navigation and wireless communication.

## 4 Experimental Section

### Experimental Setup

As depicted in Figure 2a, the piezoelectric pillars are spaced by 0.5 mm air gaps, resulting in a lattice constant of 5.5 mm. Two flexible printed circuits (FPC) are positioned on the top and bottom of the piezoelectric arrays, respectively. Each FPC consists of a 12.5 um thick PI film and incorporates 20 silver electrodes. A hollow 3D printing frame secures the array and FPCs. Epoxy adhesive is used to ensure a waterproof seal between the FPCs and the frame. Electrodes on the same FPC are linked with FPC connectors, enabling connections to the varactor diode arrays and FPGA outputs. Six varactor diodes (BB910) are connected in parallel to serve as the external capacitances for a single channel, i.e., five meta‐atoms (Figure S5, Supporting Information). These varactor diodes offer continuously variable capacitances (Figure 2c), facilitating 7‐bit phase modulation with 128 distinct reflection states. This makes them a superior choice compared to PIN diodes, which are commonly used in space‐time modulation systems but are limited to two switching states (equivalent to short or open circuits). A FPGA hardware control board based on the AMD Kintex UltraScale+ generates the 20‐channel control voltages according to pre‐designed space‐time phase function. The control voltage is updated at a rate of 20 kHz (i.e., τ  =  50 us) to achieve discrete reflection phase states, allowing for a maximum frequency modulation of 10 kHz. The typical power consumption of the FPGA is ≈10 W, while the 20‐channel DAC with amplifier circuits consumes ≈3.8 W. Consequently, the total power consumption of the operating STAM is ≈13.8 W. Reducing the pulse width of the steady phase state would increase the modulation rate, allowing for a broader range of reflected wave frequencies. However, this must be maintain the slow temporal modulation conditions to ensure accurate reflection phases, which imposes a constraint on τ. The modulation period should not be less than 8 times the 6.2 us period of the incident waves (see simulation results in Note S7, Supporting Information). For stochastic space‐time modulation, phase‐state sequences with a length of 90 000 (4.5 s) are periodically applied to each channel. The pulse width *T* for single space‐time modulation is set at 1.5 ms, comprising 30 distinct reflection phase states to ensure effective mode conversion.

### Experimental Measurements

The experimental measurements are conducted in a 1.5 × 1 × 0.7 m anechoic tank with inner walls covered with mute wedges, providing a cutoff frequency of 50 kHz (see experimental setup in Note S4, Supporting Information). At the operation frequency of 162 kHz, acoustic waves are effectively absorbed, creating a near free‐field underwater environment. A piezoelectric transducer (DYW‐150‐E) with a center frequency of 150 kHz serves as the sound source, and a hydrophone (B&K Type 8130) is used as the detector. A collaborative robot (AUBO‐i5), with an accuracy of 0.02 mm, is used to control either the transducer (in experiments involving chirp modulation and stochastic space‐time modulation for DOA estimation) or the hydrophone (in deterministic space‐time modulation experiments) using 3D printing fixtures. In the former setup, the transducer is positioned along a circular path with a radius of 20 cm from the center of the STAM, while the hydrophone is placed 14 cm below the water surface. In the latter configuration, the transducer is placed 20 cm below the water surface, and the hydrophone is situated along a circular path with a radius of 14 cm from the STAM. Both the source and detector meet the far‐field conditions. The emitting and receiving sound signals are analyzed by a multifunction I/O module (National Instruments PXI‐6115).

### Modelling of the meta‐atom

From Figure 2b, the mechanical surface impedance of the meta‐atom oriented toward water with the opposite side of air boundary condition can be derived as:

(4) $$Z_{m}\;\left(\omega \right)=Z_{1}+\frac{1}{\frac{1}{Z_{2}-N^{2}\frac{C_{v}}{j\omega C_{0}\left(C_{0}+C_{v}\right)}}+\frac{1}{Z_{1}+Z_{r}}}$$

Consequently, the reflection coefficient Γ(ω) of the meta‐atom is expressed as:

(5) $$\Gamma \;\left(\omega \right)=\frac{Z_{m}\left(\omega \right)/S-Z_{w}}{Z_{m}\left(\omega \right)/S+Z_{w}}\;$$

Here, $Z_{1}=j\rho vS\tan\frac{kL}{2}$ and $Z_{2}=R+\frac{\rho vS}{j\sin kL}$ denote different parts of mechanical impedance, $Z_{r}\approx \frac{\rho_{a}c_{a}k_{a}^{2}}{2\pi }S^{2}+j\rho_{a}c_{a}S\left(\frac{8}{3\pi }k_{a}d\right)$ is the radiation impedance of the air‐facing side of the meta‐atom, and *Z*_w_ = ρ_w_ *c* is the characteristic impedance of water with the mass density of ρ_w_ =  1 g∙cm^−3^ and sound speed of *c*  =  1500 m∙s^−1^. $C_{0}=\frac{S}{L(\beta_{33}^{T}+g_{33}^{2}/s_{33}^{D})}$ and $N=\frac{g_{33}S}{s_{33}^{D}{\bar{\beta }}_{33}L}$ were respectively the clamped capacitance and electromechanically coupling coefficient of the piezoelectric pillar. Above parameters are derived from some fundamental constants, obtained via electrical experiments using an impedance analyzer (TH‐2851, see experimental details in Note S3, Supporting Information). These constants include elastic constant under short circuit conditions $s_{33}^{D}$, direct voltage coefficient *g*_33_, dielectric permittivity $\beta_{33}^{T}$, and the 2D clamped dielectric permittivity ${\bar{\beta }}_{33}=\beta_{33}^{T}\;\left(1+\frac{g_{33}^{2}}{s_{33}^{D}\beta_{33}^{T}}\right)$. In addition, *S*, *L*, and *d* are respectively the cross‐sectional area, thickness, and width of the piezoelectric element. The terms ρ, $v=\frac{1}{\sqrt{\rho s_{33}^{D}}}$, and *R* denote the mass density, sound speed, and mechanical damping of PZT‐5H. Meanwhile, ρ_a_, *c*_a_, and *k*_a_ =  ω/*c*_a_ refer to the air density, sound wave velocity, and wavenumber, respectively. Typical values of these parameters are provided in Note S8 (Supporting Information).

## Conflict of Interest

The authors declare no conflict of interest.

## Author Contributions

Y.‐H.Y. and H. J. conceived the idea; Y.‐H.Y., J.‐Y.L., and T.L. performed the analytical modeling; Y.‐H.Y. and Y.‐Z.Y. conducted sample fabrications and experiments. Y.‐H.Y., H.J., J.‐Y.L., and T.L. wrote the manuscript; J.Y. and Z.‐Y.L. supervised the research. All authors contributed to discussions of the results and the manuscript.

## Supporting information



Supporting Information

## Data Availability

The data that support the findings of this study are available from the corresponding author upon reasonable request.
