# Supplementary material for: Acoustic Metasurface for Space‐time Reflection Manipulation
Source: Adv Sci (Weinh). 2025 Jun 26;12(36):e06308. doi: 10.1002/advs.202506308 (PMC12463000; doi:10.1002/advs.202506308)
Supplement: Supplementary file 1 — Supporting Information [file ADVS-12-e06308-s001.docx]

Supplementary Information for

**Acoustic metasurface for space-time reflection manipulation**

Yunhan Yang^1,2^, Han Jia^*2,3^, Jiuyang Lu^*4^, Yuzhen Yang^1^, Tuo Liu^1,3^, Jun Yang^*1,2^ & Zhengyou Liu^*4,5^

^1^ Key Laboratory of Noise and Vibration Research, Institute of Acoustics, Chinese Academy of Sciences, Beijing 100190, China

^2^ University of Chinese Academy of Sciences, Beijing 100049, China

^3^ State Key Laboratory of Acoustics, Institute of Acoustics, Chinese Academy of Sciences, Beijing 100190, China

^4^ Key Laboratory of Artificial Micro- and Nanostructures of Ministry of Education and School of Physics and Technology, Wuhan University, Wuhan 430072, China

^5^ Institute for Advanced Studies, Wuhan University, Wuhan 430072, China

^*^ Authors to whom correspondence should be addressed: hjia@mail.ioa.ac.cn; jylu@whu.edu.cn; jyang@mail.ioa.ac.cn; zyliu@whu.edu.cn.

[**Supplementary Information for** 1](#_Toc199535493)

[Supplementary Note 1: Discrete-time phase modulation 2](#_Toc199535494)

[Supplementary Note 2: Calculations of the Direction-of-arrival (DOA) estimation 6](#_Toc199535495)

[Supplementary Note 3: Modelling of piezoelectric meta-atoms 8](#_Toc199535496)

[Supplementary Note 4: Photograph of experimental setup 10](#_Toc199535497)

[Supplementary Note 5: Experimental signal analysis and processing 11](#_Toc199535498)

[Supplementary Note 6: Correlation coefficient matrices calculation 15](#_Toc199535499)

[Supplementary Note 7: Influence of modulation frequency 17](#_Toc199535500)

[Supplementary Note 8: Table of property parameters 19](#_Toc199535501)

Supplementary Note 1: Discrete-time phase modulation

In experiments, reflection phase functions are implemented in a discrete-time form of:

$$\begin{aligned} \varphi\left( t \right)=\sum_{n} \varphi_{n}U_{n}\left( t \right), \#\left( S1 \right) \end{aligned}$$

where $\varphi_{n}$ represents the reflection phase, and $U_{n}(t)$ is a rectangular pulse function defined as $U_{n}\left( t \right)=\left\{ \begin{aligned} 1, \left( n-1 \right)\tau<t<n\tau\\ 0, \mathrm{otherwise} \end{aligned} \right.$. Here, $\tau$ denotes the pulse width of $\varphi_{n}$, which corresponds to the duration of a steady-phase state. In our demonstrations, the switching frequency of the steady-phase state is 20 kHz, corresponding to $\tau$ of $50$us. We employ a universal discretization strategy with a time step of $\tau$ to encode not only a single period of the harmonic phase function, which has a time scale of microseconds, but also to discretize the entire modulation process over several seconds, encompassing thousands of distinct harmonic modulations. However, the discrete-time pulse function induces harmonics due to spectrum leakage. To assess the impact of discrete-time phase modulation, we compute the amplitudes of harmonics reflected by the space-time acoustic metasurface (STAM) under different space-time variables and phase precisions. The reflection phase function $\varphi(t)$ is set as a periodic space-time function featuring a period of $T_{0}$, which induces a fundamental frequency shift of $\Delta\omega=1/T_{0}$. The far-field directivity of the *m*th harmonic frequency $\omega_{0}+m\Delta\omega$ is expressed as follows^1,2^:

$$\begin{aligned} D^{\left( m \right)}\left( \theta\right)=\frac{\Gamma}{L}\cos\theta\sum_{p=1}^{N} \sum_{n=1}^{L} \mathrm{sinc}\left( \frac{\pi m}{L} \right)\cdot e^{\left[ -j\left( 2n-1 \right)\frac{\pi m}{L}+j\varphi_{n}+2\pi j\left( p-1 \right)d\left( \frac{\sin\theta_{i}}{\lambda_{i}}+\frac{\sin\theta}{\lambda_{r}^{\left( m \right)}} \right) \right]}\#\left( S2 \right) \end{aligned}$$

where $p$ denotes the spatial index of the meta-atom, $N$ is the total number of the meta-atom, $\Gamma=1$ indicates the identical reflection amplitude, $L$ is the length of the phase sequence in a single modulation period, $\lambda_{i}=2\pi\cdot c/\omega_{0}$ is the wavelength of the incident waves, $\lambda_{r}^{(m)}=2\pi\cdot c/(\omega_{0}+m\Delta\omega)$ is the wavelength of the *m*th reflected wave, $\theta_{i}$ is the incident angle, $\omega_{0}=162\times2\pi kHz$ is the operating frequency, and $c=1500 m\cdot s^{-1}$ denotes the sound speed of water. The phase sequence length $L=T_{0}/\tau$ is determined by the modulation period $T_{0}$ and the steady-state duration $\tau$. In the following calculations, a modulation period of $T_{0}=500$ us is used (i.e., $\Delta\omega=2\times2\pi\mathrm{kHz}$), yielding $L=10$.

We present calculations for two modulation scenarios: (I) time modulation with a theoretical phase function of $\varphi_{1}\left( t \right)=-\Delta\omega t$, and (II) deterministic space-time modulation with a theoretical phase function of $\varphi_{2}\left( t \right)=-\Delta\omega t-\Delta kx$, where the momentum shift $\Delta k$ is specified as 339.3 m^-1^. Both modulation types are designed to concentrate energy at the -1st harmonic frequency, with target reflection angles of 0° for scenario I and 30° for scenario II. Meanwhile, we assess the impact of phase precision by comparing two different phase coding bit depths: a higher precision of 7 bits (used in the actual STAM) and a lower precision of 2 bits. These coding bits indicate the number of discrete states uniformly divided within the $2\pi$ phase range, i.e., a total of 128 states for the former and 4 states for the latter. Figure S1 illustrates the space-time-coding matrices (left column of Fig. S1) and the corresponding scattering patterns of harmonics from -2nd to +2nd (right column of Fig. S1). As illustrated in the calculated scattering patterns of time modulation (Figs. S1(b) and S1(d)), both 2-bit and 7-bit modulations concentrate dominant energy in the target -1st harmonics, which validates the effectiveness of the discrete-time phase modulation. Compared to the low-bit scenario, high-bit phase modulations provide better harmonic suppression due to the reduction of the quantization errors. For the space-time modulations, the target reflection harmonic at 30° is precisely achieved (Figs. S1(f) and S1(h)). This indicates that the transverse dimensions of the meta-atom satisfy the subwavelength condition, thus effectively imparting momentums to the incident waves.


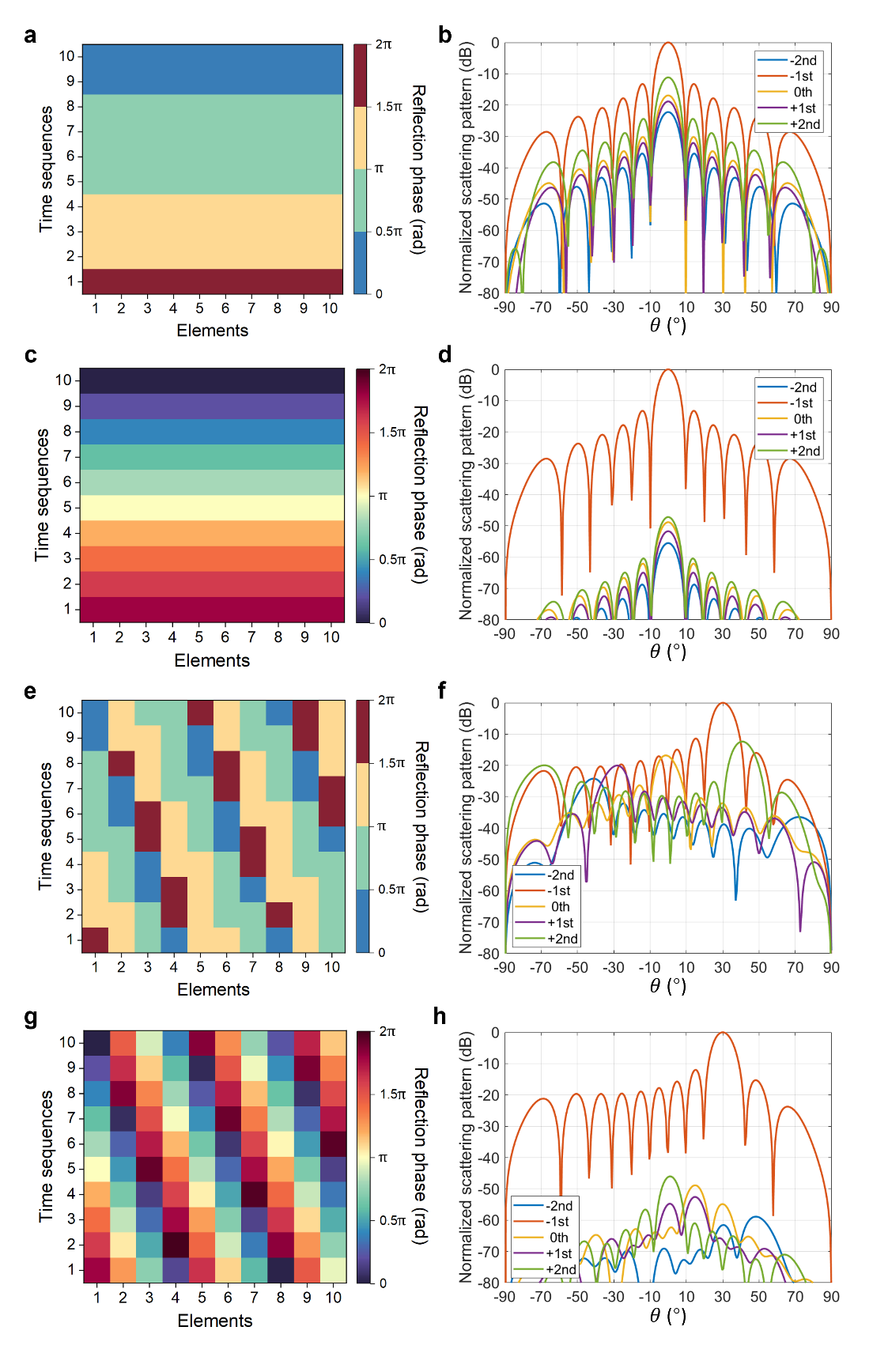


**Fig. S1 Space-time phase matrices and scattering patterns of different harmonics.** (a) Phase matrix and (b) scattering patterns under 2-bit time modulation. (c) Phase matrix and (d) scattering patterns under 7-bit time modulation. (e) Phase matrix and (f) scattering patterns under 2-bit space-time modulation. (g) Phase matrix and (h) scattering patterns under 7-bit space-time modulation.

To further investigate the influence of the number of cells in the metasurface, we present calculated scattering patterns under 7-bit space-time modulation with varying cell counts. We designed a double-beam deflection phase function: $\varphi\left( x,t \right)=\mathrm{Arg}[e^{j\left( -\Delta\omega t-\Delta kx \right)}+e^{j\left( +\Delta\omega t+1/2\Delta kx \right)}]$. This space-time phase function introduces two traveling-wave modulations to the normally incident wave, aiming to deflect the -1st harmonic wave to 30° and the +1st harmonic wave to -14.5°. As Fig. S2 depicted, both metasurfaces exhibit precise control of the reflected waves, with the 20-cell metasurface demonstrating higher directivity and a smaller full width at half maximum (FWHM). The 10-cell metasurface shows a FWHM of 11.5° for the +1st harmonic wave and 13.7° for the -1st harmonic wave, while the 20-cell metasurface exhibits a FWHM of 6.2° for the +1st harmonic wave and 7.0° for the -1st harmonic wave. These results validate the approach of increasing the number of cells in the STAM to achieve more accurate wave control.


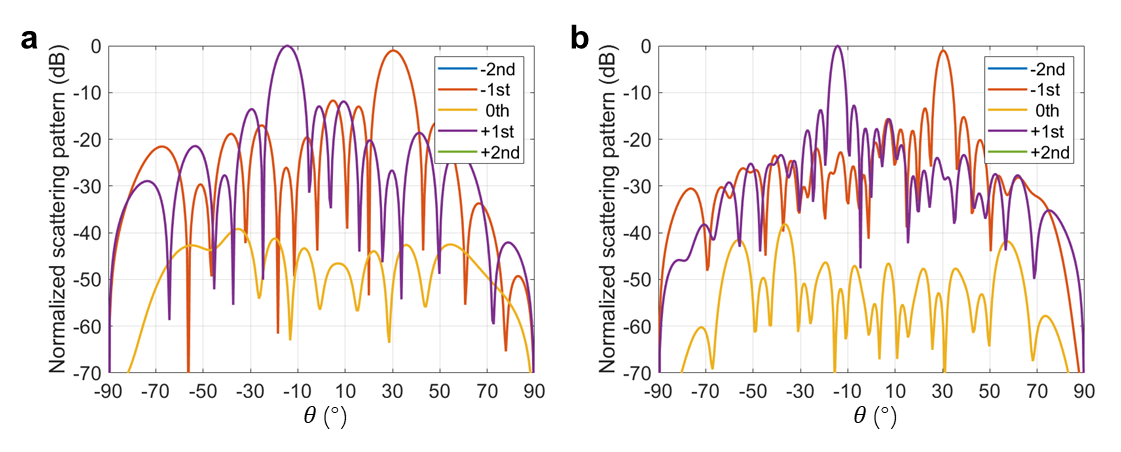


**Fig. S2 Scattering patterns of harmonics for metasurfaces with varying cell numbers.** (a) Scattering patterns of 10-cell metasurface. (b) Scattering patterns of 20-cell metasurface.

Supplementary Note 2: Calculations of the Direction-of-arrival (DOA) estimation

Under stochastic space-time modulation with a conversion slope of $c_{u}$, the incident mode with an angle of $\theta_{i}$ is converted to a lineshape distribution of reflected modes in the $k_{x}-\omega$ space (Fig. S3), where $k_{0}=\omega_{0}/c$ denotes the wavevector of the incident mode. The reflected mode line is thus expressed as:

$$\begin{aligned} \omega=-c_{u}k_{x}+\omega_{0}-c_{u}\sin\theta_{i}k_{0}.\#\left( S3 \right) \end{aligned}$$

The detected spectrum line, representing the measured spectrum at an angle $\theta_{D}$, is given as:

$$\begin{aligned} \omega=\frac{c}{\sin\theta_{D}}k_{x}\#\left( S4 \right) \end{aligned}$$

Combing Eq. S3 and Eq. S4, the coordination of the intersection $(k_{c},\omega_{c})$ are obtained:

$$\begin{aligned} k_{c}=\frac{\omega_{0}-c_{u}k_{0}\sin\theta_{i}}{\frac{c}{\sin\theta_{D}}+c_{u}},\#\left( S5 \right) \end{aligned}$$

$$\begin{aligned} \omega_{c}=\omega_{0}\frac{c-c_{u}\sin\theta_{i}}{c+c_{u}\sin\theta_{D}},\#\left( S6 \right) \end{aligned}$$

This intersection indicates that each incident mode, characterized by a specific incident angle $\theta_{i}$, appears as a unique frequency in the detected spectrum. Consequently, direct DOA estimation is achieved by mapping the incident angle $\theta_{i}$ to the corresponding detected frequency $\omega_{c}$:

$$\begin{aligned} \theta_{i}\left( \omega_{c} \right)=\arcsin\left[ \frac{c}{c_{u}}-\frac{\omega_{c}}{\omega_{0}}\left( \frac{c}{c_{u}}+\sin\theta_{D} \right) \right]\#\left( S7 \right) \end{aligned}$$

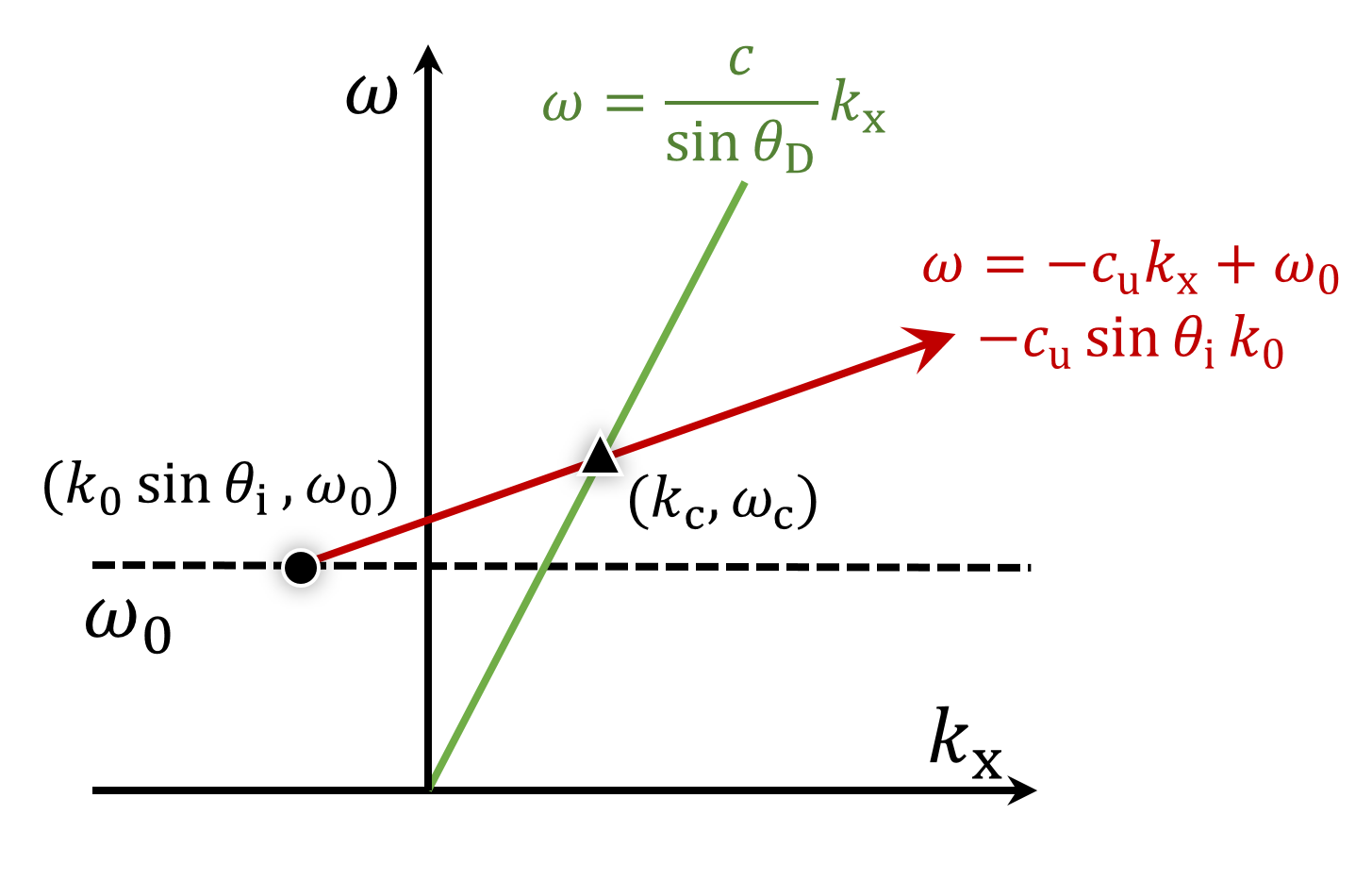


**Fig. S3 Schematic of DOA estimation in the** $\boldsymbol{k}_{\mathbf{x}}\boldsymbol{-\omega}$ **space.** The dashed line denotes the frequency of the incident waves. The green line represents the detected spectrum line at a specific angle $\theta_{D}$. The black triangle denotes the intersection $(k_{c},\omega_{c})$. The red arrow line depicts the distribution of the reflected modes, while the arrow indicating the modulation direction.

Supplementary Note 3: Modelling of piezoelectric meta-atoms

The parameters of the piezoelectric meta-atoms used in the main text are extracted from electrical experiments with an impedance analyzer (TH-2851). To elucidate this process, we depict the equivalent circuit of the piezoelectric unit under electrical port excitations in Fig. S4.


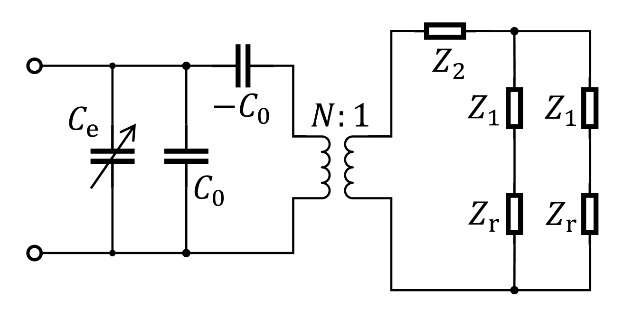


**Fig. S4 Equivalent circuit of the piezoelectric unit under electrical excitation.**

In this context, the electrical impedance of the meta-atom with an external capacitance $C_{e}$ is given by:

$$\begin{aligned} Z_{e}\left( \omega\right)=\frac{1}{\frac{1}{\left[ \left( \frac{1}{2}Z_{1}+\frac{1}{2}Z_{r}+Z_{2} \right)\frac{1}{N^{2}}-\frac{1}{j\omega C_{0}} \right]}+j\omega C_{0}+j\omega C_{e}},\#\left( S8 \right) \end{aligned}$$

where all parameters included are consistent with the scenarios under mechanical excitations, which is described in Methods in the main text. We select capacitors with a variety of capacitances to modify external capacitance ($C_{e}$), specifically 0 (open circuit), 10.7, 22.0, 42.7, 89.1, 143.4, and 244.5 pF. It is worth noting that each channel consists of 5 parallel piezoelectric pillars. Consequently, for each pillar, the averaged external capacitances are 0, 2.1, 4.4, 8.5, 17.8, 28.7, and 48.9 pF, respectively.

With an impedance analyzer, we measure the impedance curves $\left| Z_{\exp} \right|$ within a range from 100 kHz to 250 kHz for averaged piezoelectric pillars of each channel under different external capacitances and fit them with $\left| Z_{e} \right|$ using a genetic algorithm based on the damped least-squares method to extract fundamental parameters: $R$, $s_{33}^{D}$, $g_{33}$, and $\beta_{33}^{T}$. Figures S5(a-c) present the experimental impedance curves $\left| Z_{\exp} \right|$ of the averaged elements in 4th (Fig. S5(a)), 12th (Fig. S5(b)), and 20th (Fig. S5(c)). In the meanwhile, Figs. S5(d-f) and S5(g-i) illustrate the corresponding fitted impedance curves $\left| Z_{e} \right|$ and their phases $\mathrm{Arg}\left( Z_{e} \right)=\tan^{-1} \left[ \frac{\mathrm{Im}\left( Z_{e} \right)}{\mathrm{Re}\left( Z_{e} \right)} \right]$, respectively. It can be observed from the figures that the impedance curves across different channels are nearly identical. This demonstrates good consistency in the sample fabrication. As the external capacitance $C_{e}$ increases, the peak frequencies of the electrical impedance curves illustrate explicit red shifts, while the dips remain unchanged. These electrical impedance peaks are related to the resonance frequencies, aligning with the results in Figs. 2d and 2e in the main text. The remarkable consistency between the fitted impedance curves and the measured results validates the theoretical model and the extracted parameters.


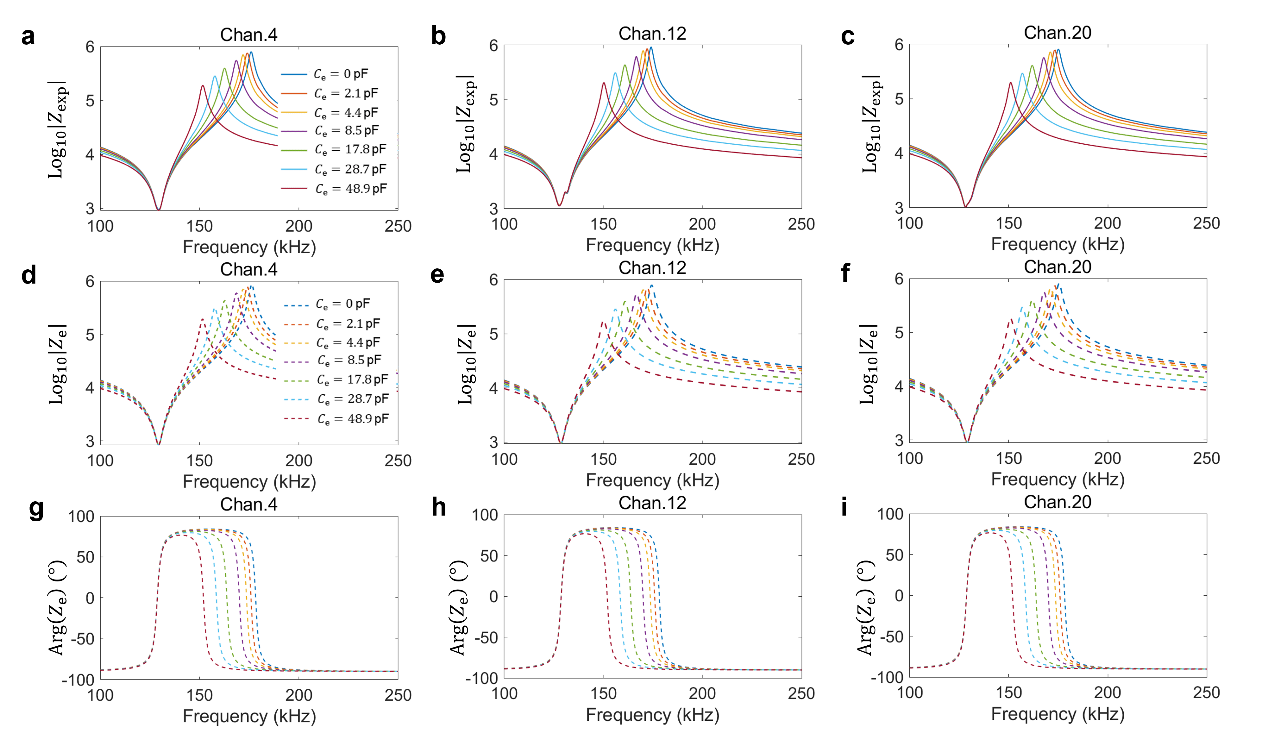


**Fig. S5** (a-c) Measured electrical impedance curves, (d-f) fitted electrical impedance curves, and (g-i) fitted electrical phases of averaged meta-atom for channel 4 (a, d, g), 12 (b, e, h), and 20 (c, f, i).

Supplementary Note 4: Photograph of experimental setup


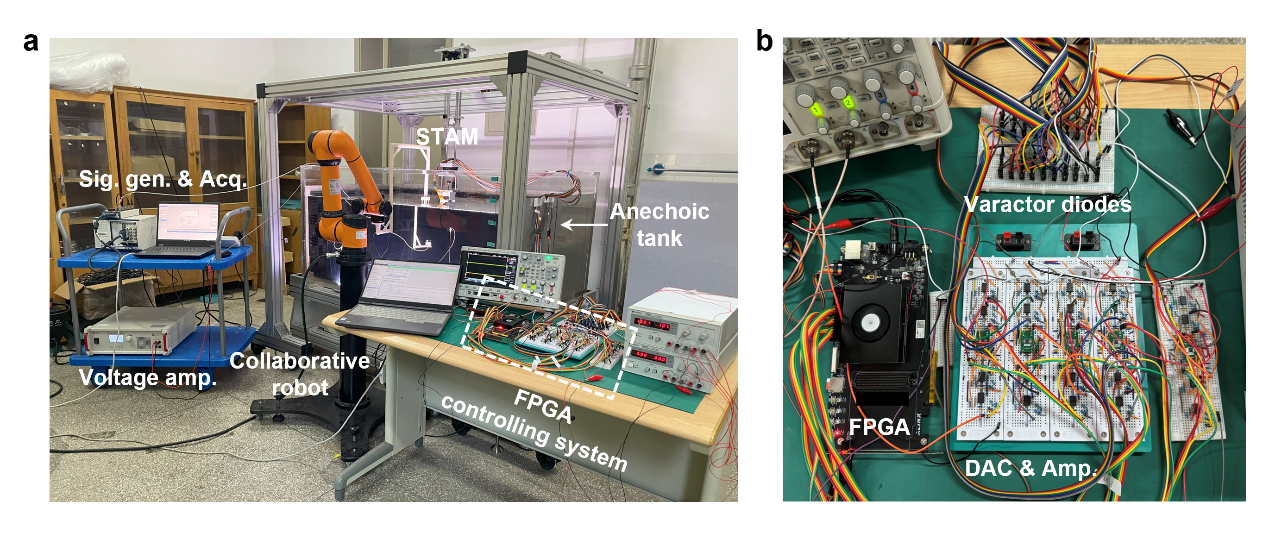


**Fig. S6 Experimental setup.** (a) Measurement configuration, including an anechoic tank, a signal generation and acquisition module, a collaborative robot, and the STAM controlled by a field-programmable gate array (FPGA) system. (b) The FPGA controlling system, involving a FPGA hardware control board, digital-to-analog converters (DAC) with amplifier circuits, and a 20-channel array of BB910 varactor diodes with 6 diodes per channel.

Supplementary Note 5: Experimental signal analysis and processing

In experiments, the source is continuously generating sinusoidal signals at the operating frequency of 162 kHz and the sound signals are acquired by the I/O module at a frequency of 500 kHz. Figure S7(b) presents the power spectrum density (PSD) obtained by the hydrophone without reflection modulation on the STAM (measurement setup is depicted in Fig. S7(a)), revealing a typical line spectrum with energy concentrated at the operating frequency of 162 kHz. Subsequently, the STAM is configured with a phase function $\varphi\left( t \right)=\omega_{m}t$ to perform time modulation, where $\omega_{m}=3.5\times2\pi$ kHz. Figures S7(c) and S7(d) present the measured PSD for the time modulation experiment, where the reflected waves show accurate blue-shift from 162 kHz to 165.5 kHz. It is worth noting that, the original frequency component 162 kHz persists with high energy, attributed to the measured spectrum encompassing both incident/direct and reflected waves. The direct waves travel a mere 6 cm from the source to the hydrophone, whereas the reflected waves cover a distance of 34 cm to be detected, resulting in the reflected waves being reasonably weaker than the direct waves. Additionally, a frequency component at 169 kHz is observed, corresponding to the +2nd-order harmonics due to phase matching.


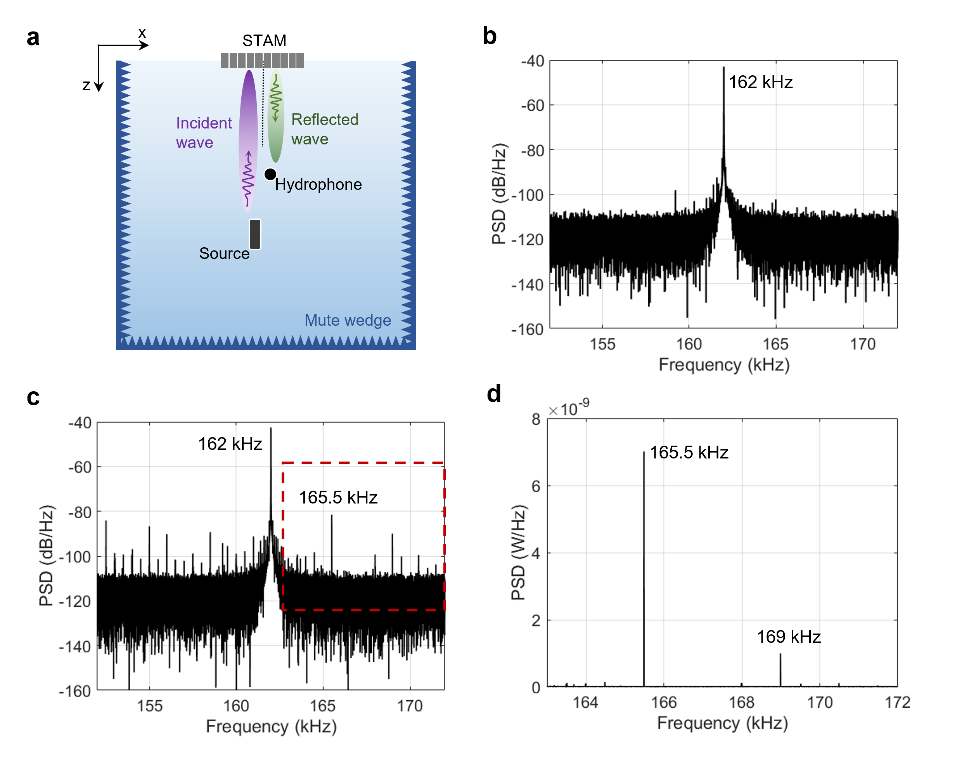


**Fig. S7 Measured spectra of the source and normal incidence with time modulation.** (a) Schematic of the measurement setup. (b) PSD of the source. (c) PSD under time modulation. (d) Enlarged detail of the PSD shown in (c).

To further illustrate the spectra obtained in experiments involving deterministic space-time modulation, we extract the measured spectrum under conditions of deterministic mode conversion slope and frequency shift ($c_{\mathrm{st}}^{(2)}$ and $\omega_{\mathrm{st}}^{(2)}$ in the main text). In this setup, the hydrophone is positioned at an angle of -23° relative to the STAM. The measured spectra depicted in Figs. S8(b) and S8(c) clearly demonstrate a pronounced component at the target reflection frequency of 165.9 kHz. Compared with the case of time modulation, the spectra from the deterministic space-time modulation exhibit more concentrated energy at the target frequency and better subharmonics suppression. This is attributed to the challenge of satisfying phase-matching condition for harmonics in experiments involving both space-time modulation, leading to the subharmonics of the reflected waves being redirected.


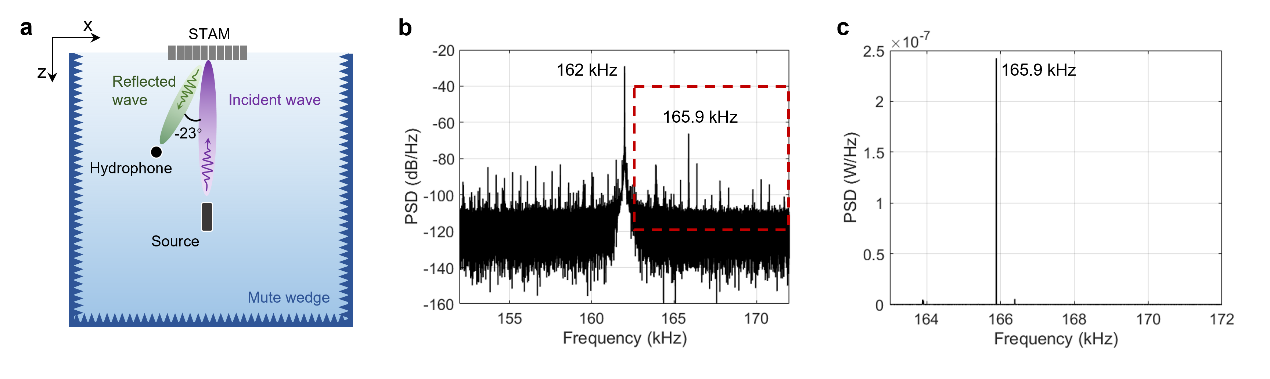


**Fig. S8 Measured spectra of normal incidence with deterministic space-time modulation.** (a) Schematic of the measurement setup. (b) PSD under deterministic space-time modulation. (c) Enlarged detail of the PSD shown in (b).

In stochastic space-time modulation experiments, each measurement’s total acquisition time is set as 5 seconds. This leads to a high-resolution spectrum with a spectral resolution of 0.2 Hz. For virtualization, we extract the envelopes of the spectra using a moving average smoothing method with a window length of 200 Hz. Figure S8 presents the original spectra (black curves) and smoothed spectra (red curves) from both down-conversion (Figs. S9(a-c)) and up-conversion (Figs. S9(d-f)) experiments. The left column displays the results of down-conversion mapping, while the right column shows the up-conversion mapping. Clear frequency shifts are observed as the incident angle varies, and the envelopes facilitate characterization.


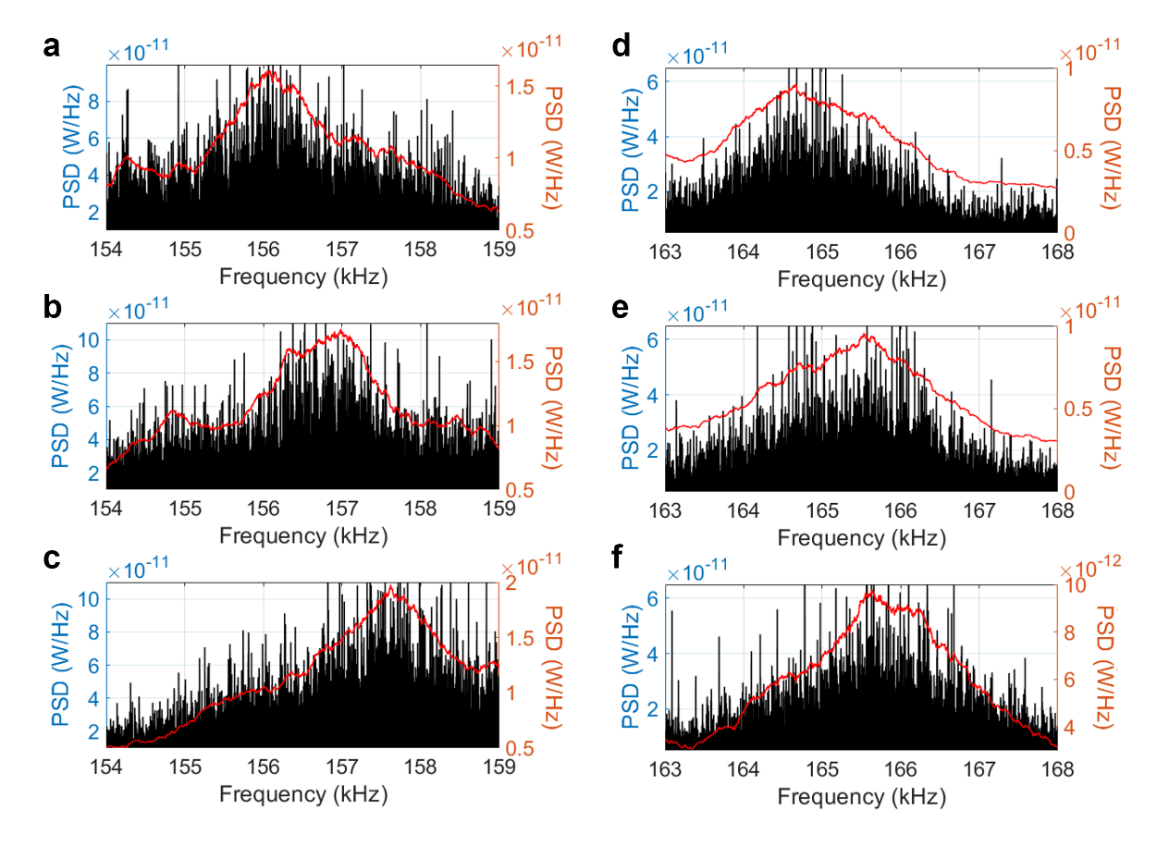


**Fig. S9 Measured PSD in the experiments of stochastic space-time modulation.** (a-c) Measured PSD (black lines) and extracted envelopes (red lines) of the stochastic space-time modulation experiments with down-conversion modulation. The incident angles are (a) 12.5°, (b) 7.5°, and (c) 2.5°, respectively. (d-f) Measured PSD (black lines) and extracted envelopes (red lines) of the stochastic space-time modulation experiments with up-conversion modulation. The incident angles are (d) -10°, (e) -5°, and (f) 0°, respectively.

Supplementary Note 6: Correlation coefficient matrices calculation

To quantify the quality of the DOA estimation across different incidence channels, we introduce the correlation coefficient $\rho_{i,j}$, defined as follows^3,4^:

$$\begin{aligned} \rho_{ij}=\frac{\mathrm{cov}\left( T_{i},M_{j} \right)}{\sqrt{D\left( T_{i} \right)\cdot D\left( M_{j} \right)}},\#\left( S9 \right) \end{aligned}$$

where $T_{i}$ and $M_{j}$ represent the $i$th-channel target and $j$th-channel measured spectrum, respectively. The term $\mathrm{cov}(T_{i},M_{j})$ denotes the covariance between $T_{i}$ and $M_{j}$, while $D(T_{i})$ and $D(M_{j})$ are the variances of $T_{i}$ and $M_{j}$. For each channel, the target spectrum is modeled using a normalized Gaussian lineshape given by^5^:

$$\begin{aligned} g\left( f \right)=e^{-\frac{\left( f-f_{c} \right)^{2}}{2\sigma^{2}}},\#\left( S10 \right) \end{aligned}$$

where $\sigma$ represents the standard variance, corresponding to the FWHM of the curve as per the relationship $\mathrm{FWHM}=2\sqrt{2ln2}\sigma$; $f_{c}$ denotes the theoretical peak frequency derived from Eq. S7. For our analysis, we use an FWHM of 3 kHz, resulting in $\sigma=6.3\times{10}^{3}$. Figure S9 presents the measured spectra and the corresponding target spectra from the stochastic space-time modulation experiments. The measured lineshapes observed in each channel align well with the target results, corroborating the findings presented in Fig. 4 of main text.


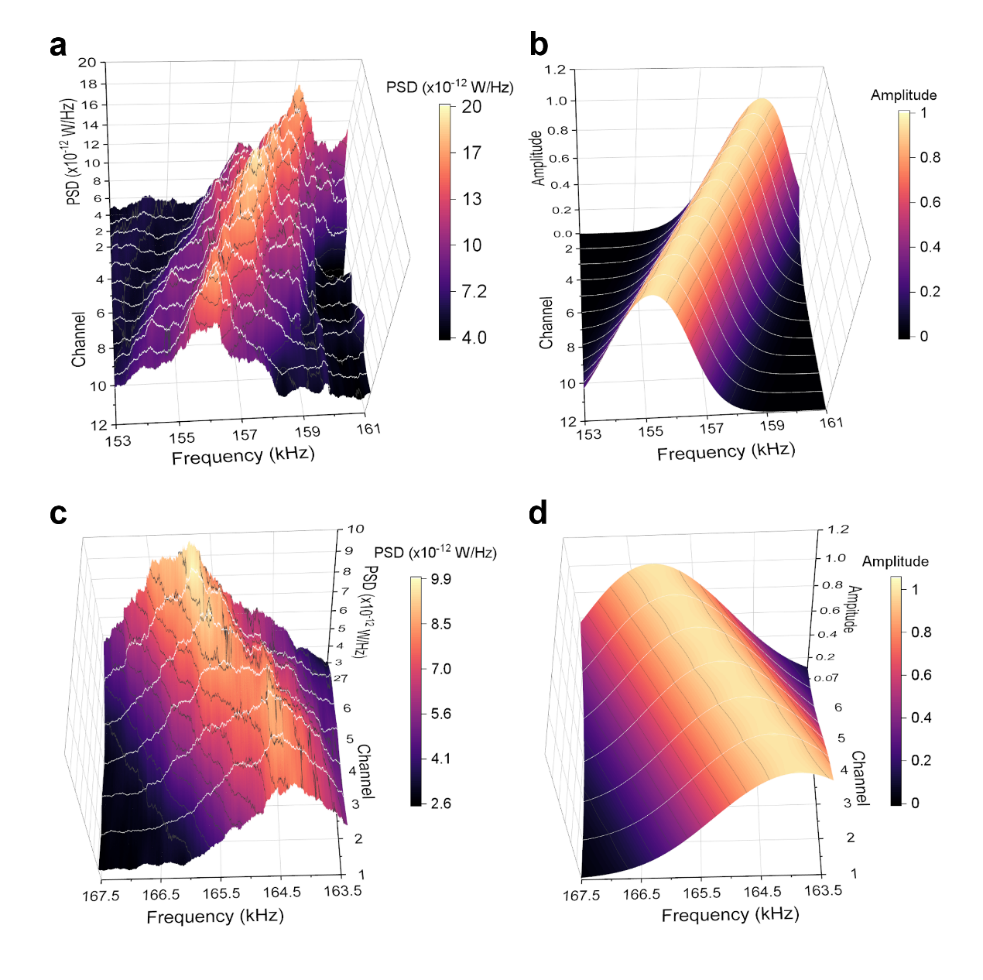


**Fig. S10 Measured and target spectra used for correlation coefficient calculation.** (a) Measured and (b) target PSD of the down-conversion modulation. (c) Measured and (d) target PSD of the up-conversion modulation.

Supplementary Note 7: Influence of modulation frequency

The space-time metasurface necessitates a modulation period significantly longer than that of the incident waves to achieve phase-matching modulation. In our experiments, we implemented a steady-state discrete-time modulation of 50 us, approximately eight times the period of the incident wave.

To investigate the influence of modulation frequency, we employed the COMSOL Pressure Acoustics Transient Module for simulation. This simulation utilized a two-dimensional model of a temporally varying impedance area to represent the discrete-time reflection phase unit in contact with water. The model matched the dimensions of the piezoelectric pillar section, measuring $5\times10$ mm, with all other boundaries set as hard. The sound speed in this area is defined in a discrete-time form as $c\left( t \right)=\sum_{n} \frac{L}{2\pi T_{0}\varphi_{n}}U_{n}\left( t \right)$, where $T_{0}\approx6.2$ us represents the period of the incident wave at an operating frequency of 162 kHz. The density of the modulating area is expressed as $\rho\left( t \right)=\rho_{0}\frac{c_{0}}{c(t)}$ to k to ensure impedance matching, with $\rho_{0}$ and $c_{0}$ denoting the density and sound speed of water, respectively.

We applied a 2-bit phase modulation, where the discrete phases $\varphi_{n}$ are 0, $\frac{\pi}{2}$, $\pi$, $\frac{3}{2}\pi$. The time-varying phase function is defined linearly as $\varphi\left( t \right)=-\omega_{m}t$, resulting in a redshift in frequency. The pulse width of $\varphi_{n}$ is denoted as $\tau$, which we used to vary the modulation frequency, with the modulation period $T=4\tau$. We collected the sound pressure of the scattering field and performed a fast Fourier transform (FFT) to obtain the modulated spectrum. The transient simulation used a time step of 2 us over a total duration of 5 ms, yielding an upper frequency limit of 250 kHz and a resolution of 200 Hz.

We set six values for $\tau$ to generate six distinct modulation frequencies. The modulation periods were ${T=T}_{0}, 2T_{0},4T_{0},8T_{0},12T_{0},20T_{0}$, corresponding to reflection wave frequencies of $f=0, 81, 121.5, 141.8, 148.5, 153.9$ kHz, respectively. The PSD spectra of the reflected waves for these scenarios are presented in Fig. S11. The upper section shows relatively fast time modulation $T{=T}_{0}, 2T_{0},4T_{0}$, while the lower section illustrates slow temporal modulation periods of $T{=8T}_{0}, 12T_{0},20T_{0}$.

In Fig. S11a, all three PSDs exhibit a significant component at the incident wave frequency of 162 kHz, indicating the absence of phase-matching conditions. For $T=4T_{0}$, the target frequency 121.6 kHz begins to dominate, indicating an improvement in phase-matching. As shown in Fig. S11b, when the modulation period extends to $T=8T_{0}$, the target frequency of 141.8 kHz becomes the primary lobe, demonstrating a high-quality frequency shift that approaches experimental conditions. Further extending the modulation period to $T={12T}_{0}$ and $20T_{0}$ maintains ideal frequency shifts. These results clearly delineate the boundaries of slow temporal modulation and validate our experimental setup.


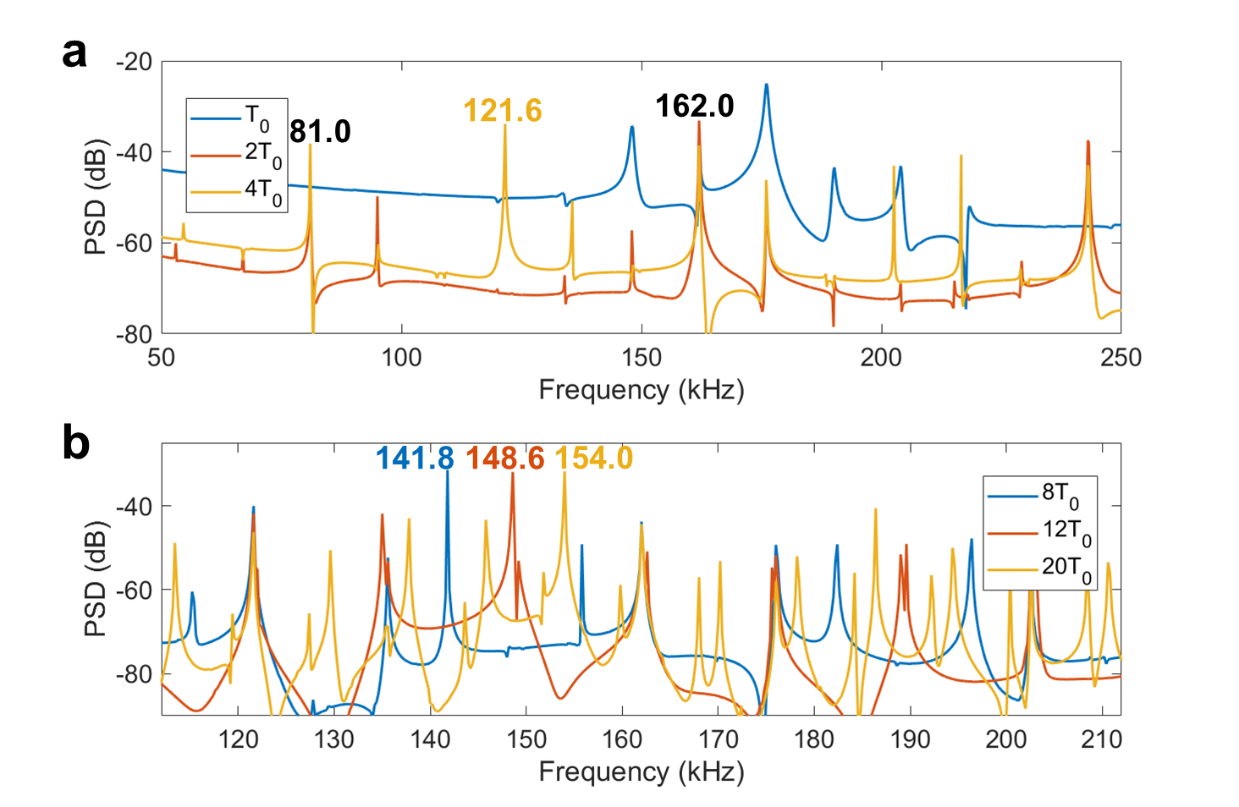


**Fig. S11 Simulated PSD of reflected waves under different modulation frequencies.** (a) PSD for scenarios of $T{=T}_{0}, 2T_{0},4T_{0}$. (b) PSD for scenarios of $T{=8T}_{0}, 12T_{0},20T_{0}$

Supplementary Note 8: Table of property parameters

|  | Property | value | Unit |  | Property | value | Unit |
| --- | --- | --- | --- | --- | --- | --- | --- |
| 1 | $s_{33}^{D}$ | 10.6 | ×10^-12^ m^2^/N | 6 | $d$ | 5 | mm |
| 2 | $\beta_{33}^{T}$ | 3.6 | ×10^7^ m/F | 7 | *S* | 2.5 | ×10^-5^ m^2^ |
| 3 | $g_{33}$ | 20.1 | ×10^-3^V∙m/N | 8 | $L$ | 10 | mm |
| 4 | $R$ | 3.5 | Ω | 9 | $\rho_{a}$ | 1.21 | kg/m^3^ |
| 5 | $\rho$ | 7.6 | g/cm^3^ | 10 | $c_{a}$ | 343 | m/s |

**References**

1. Zhang, L. *et al.* Space-time-coding digital metasurfaces. *Nat. Commun.* **9**, 4334 (2018).

2. Zhang, L. *et al.* Breaking Reciprocity with Space-Time-Coding Digital Metasurfaces. *Adv. Mater.* **31**, e1904069 (2019).

3. Sun, X. *et al.* Sound Localization and Separation in 3D Space Using a Single Microphone with a Metamaterial Enclosure. *Adv. Sci.* **7**, 1902271 (2020).

4. Xiong, B. *et al.* Breaking the limitation of polarization multiplexing in optical metasurfaces with engineered noise. *Science* **379**, 294–299 (2023).

5. Demtroeder, W. *Laser Spectroscopy 1: Basic Principles*. (Springer, New York, 2014).
